# Characterization of Ricin and *R. communis* Agglutinin Reference Materials

**DOI:** 10.3390/toxins7124856

**Published:** 2015-11-26

**Authors:** Sylvia Worbs, Martin Skiba, Martin Söderström, Marja-Leena Rapinoja, Reinhard Zeleny, Heiko Russmann, Heinz Schimmel, Paula Vanninen, Sten-Åke Fredriksson, Brigitte G. Dorner

**Affiliations:** 1 Biological Toxins, Centre for Biological Threats and Special Pathogens, Robert Koch Institute, Seestr. 10, 13353 Berlin, Germany; worbss@rki.de (S.W.); skibam@rki.de (M.S.); 2 VERIFIN (Finnish Institute for Verification of the Chemical Weapons Convention), Department of Chemistry, University of Helsinki, A.I. Virtasen aukio 1, Helsinki 05600, Finland; martin.soderstrom@helsinki.fi (M.S.); marja-leena.rapinoja@helsinki.fi (M.-L.R.); paula.vanninen @helsinki.fi (P.V.); 3 European Commission, Joint Research Centre, Institute for Reference Materials and Measurements, Retieseweg 111, 2440 Geel, Belgium; reinhard.zeleny@ec.europa.eu (R.Z.); heinz.schimmel@ec.europa.eu (H.S.); 4 Bundeswehr Research Institute for Protective Technologies and NBC Protection, Humboldtstr. 100, 29633 Munster, Germany; heikorussmann@bundeswehr.org; 5 FOI, Swedish Defence Research Agency, CBRN Defence and Security, Cementvagen 20, 901 82 Umeå, Sweden; sten-ake.fredriksson@foi.se

**Keywords:** proficiency test, ricin, reference material

## Abstract

*Ricinus communis* intoxications have been known for centuries and were attributed to the toxic protein ricin. Due to its toxicity, availability, ease of preparation, and the lack of medical countermeasures, ricin attracted interest as a potential biological warfare agent. While different technologies for ricin analysis have been established, hardly any universally agreed-upon “gold standards” are available. Expert laboratories currently use differently purified in-house materials, making any comparison of accuracy and sensitivity of different methods nearly impossible. Technically challenging is the discrimination of ricin from *R. communis* agglutinin (RCA120), a less toxic but highly homologous protein also contained in *R. communis*. Here, we established both highly pure ricin and RCA120 reference materials which were extensively characterized by gel electrophoresis, liquid chromatography-electrospray ionization-tandem mass spectrometry (LC-ESI MS/MS), and matrix-assisted laser desorption ionization–time of flight approaches as well as immunological and functional techniques. Purity reached >97% for ricin and >99% for RCA120. Different isoforms of ricin and RCA120 were identified unambiguously and distinguished by LC-ESI MS/MS. In terms of function, a real-time cytotoxicity assay showed that ricin is approximately 300-fold more toxic than RCA120. The highly pure ricin and RCA120 reference materials were used to conduct an international proficiency test.

## 1. Introduction

Originally identified by Stillmark in 1888 [1], ricin is produced by *Ricinus (R.) communis* and is one of the most toxic plant toxins known today. It belongs to the family of type II ribosome-inactivating proteins [2]. As a prototype AB toxin, ricin consists of a sugar-binding B chain (~34 kDa) linked via a disulfide bond to the catalytically active A chain (~32 kDa) which acts as an RNA *N*-glycosidase, resulting in a holotoxin of about 65 kDa [3,4]. As lectin, the B chain mediates cell binding via different oligosaccharide residues on the cell surface, including *N*-acetylglucosamine and galactose residues found on glycolipids and glycoproteins [5,6,7]. Previously, oligosaccharides have been employed for the purification of ricin by affinity chromatography [8,9,10]. After internalization, the A-B heterodimer undergoes retrograde transport via the Golgi network to the endoplasmic reticulum where the heterodimer is reduced and separated into the two subchains [11,12]. The A chain is then transported into the cytosol and binds to the ribosome where it removes a single adenine from the 28S rRNA, thus preventing further binding of elongation factors, inhibiting protein biosynthesis, and finally leading to cell death [13,14,15].

The understanding of ricin is complicated by the fact that, besides ricin, *R. communis* seeds contain the homologous but less toxic protein *R. communis* agglutinin, abbreviated RCA120 [6]. RCA120 is a 120 kDa heterotetrameric protein consisting of two ricin-like heterodimers linked via a disulfide bond between the two A chains [16]. Different isoforms of ricin have been described, adding further complexity to the issue: the original isoform now termed ricin D is accompanied in most *R. communis* cultivars by the isoform ricin E which contains a hybrid B chain composed of the *N*-terminal part of the original ricin B chain and the *C*-terminal part of the RCA120 B chain ([17,18,19]).

Ricin and RCA120 show a high sequence homology of 93% and 84% between the A and B chains of ricin and RCA120, respectively [20]. Still, ricin is a potent toxin but a weak hemagglutinin, whereas RCA120 is only a weak toxin but a strong hemagglutinin [21,22]. Depending on the experimental system used, the difference in toxicity between ricin and RCA120 was described to be about 60–2000 times [8,22,23,24]. Accidental and intended *R. communis* intoxications in humans and animals have been known for centuries. The toxicity of ricin *in vivo* is estimated to be 1–20 mg/kg body weight when ingested and 1–10 µg/kg body weight when delivered by inhalation or injection [4].

Both ricin and RCA120 are not single copy genes, but rather part of a larger ricin gene family encoding for seven full-length ricin or ricin-like proteins and several potential shorter gene products of unknown expression and function, indicating a greater variability than previously anticipated [4,25,26]. The seven full-length proteins of the ricin gene family have been found to inhibit protein synthesis similar to ricin itself [4,26]. Ricin contains four glycosylation sites, two on the A chain and two on the B chain [4,27], and additional heterogeneity of the molecule is based on different glycosylation patterns: it has been shown that variable toxicities of ricin isoforms have been correlated with different glycosylation levels [28,29]. Another level of complexity has recently been added by the description of heterogeneity in the deamidation pattern, the conversion rate of single asparagine residues to aspartic and isoaspartic acid [30].

Ricin and the ricin-producing plant are typical dual-use substances: *R. communis* is grown worldwide on an industrial scale as a source of castor oil which is—because of the high content of the unsaturated fatty acid ricinoleic acid and its favorable physico-chemical properties—a valued raw material for the production of lubricants, pharmaceuticals, cosmetics, paints, coatings, inks, and many other products. During the extraction process ricin accrues as a by-product of the oil’s production [31]. Its high toxicity, availability, and the relative ease of extraction make ricin a potential agent for bioterrorism [32]. Consequently, ricin is listed as a category B agent of potential bioterrorism risk by the Centers for Disease Control and Prevention (CDC) [33]. Actually, ricin has been used for small-scale attacks such as the assassination of Georgi Markov [34,35]. High media coverage was gained by the ricin-containing threat letters sent in 2003 and 2013 to members of the U.S. Senate and the White House as well as to U.S. President Obama [36,37]. Additionally, ricin has a history of military use by different nations and was included in different weapons programs during World War II and later [36,38,39,40]. Therefore, ricin is a prohibited substance both under the Chemical Weapons Convention (CWC, schedule 1 compound) and the Biological Weapons Convention (BWC); its possession and production must be declared to the Organisation for the Prohibition of Chemical Weapons (OPCW), and it may be used only for strictly specified purposes defined in the CWC.

Against the background of the toxin’s potential misuse for terrorist, criminal, or military purposes, the rapid, sensitive, and ideally unambiguous detection of ricin is necessary. While different technologies for ricin detection and identification have been established using immunological, spectrometric, functional, or molecular approaches, hardly any universally agreed-upon “gold standards” are available [4]. No certified reference material is available, and expert laboratories currently use differently purified in-house materials as a standard, making any comparison of accuracy and sensitivity of different methods nearly impossible. Also there are open questions as to which methods have to be used and combined to obtain preliminary, confirmed, and unambiguous results. Depending on the task and scenario, the discrimination of ricin from the homologous RCA120 is important, as only ricin is recognized as a threat agent under the BWC and a schedule 1 component under the CWC. In the context of a forensic analysis it might be important to present information on purity and amount, biological activity, and potential source of a suspect sample.

In this work, we have established both highly pure ricin and RCA120 reference materials which were extensively characterized by biochemical, spectrometric, immunological, and functional techniques. Especially liquid chromatography-electrospray ionization-tandem mass spectrometry (LC-ESI MS/MS) was instrumental in differentiating the related ricin D, ricin E, and RCA120 from each other. Both materials were later used in the framework of the European Union (EU) project EQuATox (Establishment of quality assurance for the detection of biological toxins of potential bioterrorism risk [41]) funded under the European Community’s Seventh Framework Programme to organize and conduct a large international proficiency test (PT, [42]).

## 2. Results and Discussion

Depending on the primary protein sequence and its specific glycosylation and deamidation level, ricin is a heterogeneous molecule with variable molecular weight, protein charge/isoelectric point, and toxicity [27,28,29,30]. Against this background, an important question is from which cultivar or plant variety ricin and the highly homologous RCA120 should be purified to generate well-characterized reference materials since any characteristic might be attributed to that particular toxin isolated from a selected cultivar and might not automatically be applied to toxins from other sources [27,30]. Commercially available ricin and RCA120 were purified from an unknown cultivar and also show variable purities: 96% purity for ricin was reported, but only 68% purity for RCA120 [43]. The question was discussed in the expert forum of the EQuATox consortium representing scientists from 35 laboratories and 20 countries internationally. Specifically, it was discussed whether the cultivar *R. communis* *zanzibariensis* should be used as toxin source which has been shown to contain ricin D only [44] or whether a cultivar like *R. communis* *carmencita* should be used which is known to contain both ricin D and ricin E isolectins. Ricin from *R. communis* *zanzibariensis* can be clearly differentiated from ricin purified from a panel of other cultivars and seems to be particular [27]. Therefore, it was decided to use a more representative cultivar as source of toxin, even if this meant to purify a mixture of ricin isolectins, namely D and E.

**Figure 1:**
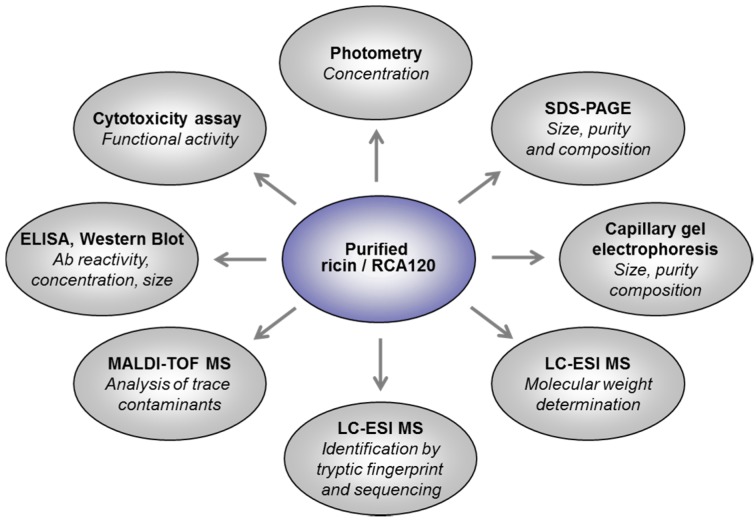
Schematic overview of technical approaches used to describe the purity, composition, and activity of highly purified ricin and RCA120 from *R. communis carmencita*.

To this end, ricin and RCA120 were prepared from the seeds of *R. communis* *carmencita* following previously published procedures [8,45,46]. Both protein preparations were thoroughly characterized, combining technically independent and complementary approaches to describe the purity, composition, and activity of both ricin and RCA120 using biochemical, spectrometric, immunological, and functional methods (Figure 1).

At the beginning of the study, a highly precise and accurate quantification of both purified protein preparations was important for the subsequent comparison of quantitative results reported in the proficiency test. It is known that different protein quantification methods such as Bradford assay [47], Lowry method [48], or amino acid analysis based on isotope dilution mass spectrometry [49] deliver variable results depending on the size and charge of the target protein, its amino acid composition, glycosylation pattern or other post-translational modifications, buffer conditions and potential impurities present in a protein sample [50,51]. Based on the facts that both protein preparations were determined to be highly pure glycoproteins (see Section 2.1 and Section 2.2) and to have available well-established extinction coefficients for ricin and RCA120, it was decided to use the measurement of absorbance at 280 nm for quantification of the protein content. Based on the absorption measured at 280 nm and according to Lambert-Beer’s law and the known extinction coefficients ε = 1.1615 mL × mg^−1^ × cm^−1^ for ricin [52] and ε = 1.17 mL × mg^−1^ × cm^−1^ for RCA120 [6], the protein concentrations were determined at *c*(ricin) = 1.51 ± 0.043 mg/mL and c(RCA120) = 1.03 ± 0.035 mg/mL.

### 2.1. Size, Purity and Composition

#### 2.1.1. Composition and Purity by Gel Electrophoresis

**Figure 2:**
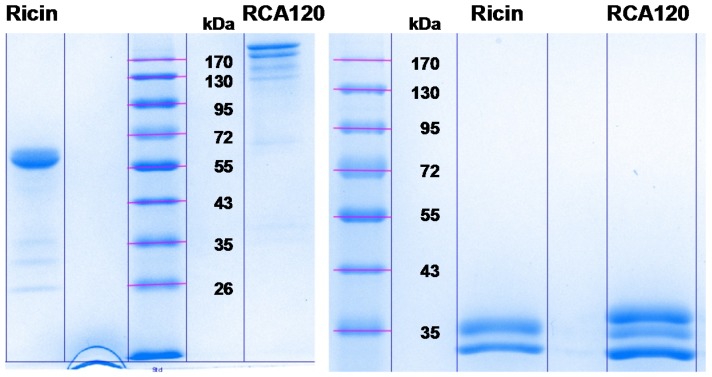
Protein separation of purified ricin and RCA120 from *R. communis carmencita*. 10% SDS-PAGE of either purified ricin (3 µg of ricin each) or purified RCA120 (5 µg of RCA120 each) separated under non-reducing conditions (left panel) or under reducing conditions (right panel, use of β-mercaptoethanol as reducing agent) followed by staining with Coomassie Brilliant Blue.

In order to display an overview of the composition and purity of the ricin and RCA120 preparations, sodium dodecyl sulfate polyacrylamide gel electrophoresis (SDS-PAGE) and capillary gel electrophoresis (CGE) were performed as a first step. As displayed in Figure 2, both ricin and RCA120 preparations showed distinct, sharp bands upon separation in an SDS-PAGE and no degradation of proteins was observed. Non-reduced ricin was running at about 60 kDa, while non-reduced RCA120 was arbitrarily running at a higher molecular weight between 120 kDa and 170 kDa. This behavior has been observed before for other preparations of RCA120 [6]. Under reducing conditions, ricin migrated in two bands of around 33 and 35 kDa, where the lower band has been shown to contain the ricin A chain and the upper band a higher glycosylated form of the ricin A chain plus the ricin B chain [51]. RCA120 was showing three bands of about 33–36 kDa, and the result was in good accord with previously reported data [6,28,29,51].

For both protein preparations, a number of faint bands of lower molecular weight than the full-length toxins were observed by SDS-PAGE. These bands could potentially represent shorter fragments of ricin or RCA120, respectively, or completely unrelated proteins co-purified from *R. communis*. The identity of these trace contaminants was clarified by matrix-assisted laser desorption ionization-time of flight mass spectrometry (MALDI-TOF MS) analysis (see Section 2.2.2).

As judged from the main protein bands, no significant cross-contamination of ricin with RCA120 or, *vice versa*, RCA120 with ricin was observed, and the purity was estimated to be >95%. Since this point was important for designing proper PT samples, it was further analyzed using CGE.

**Figure 3:**
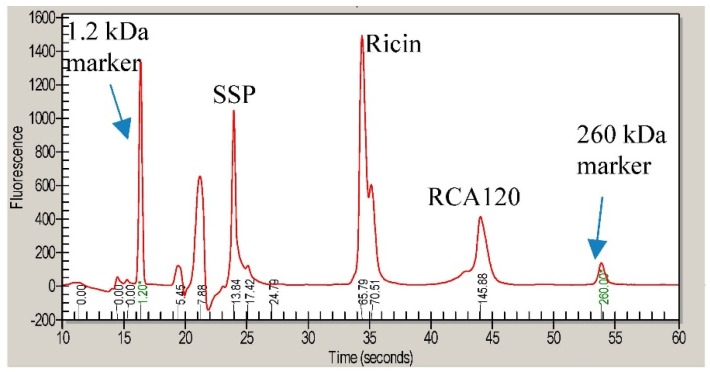
CGE analysis of a crude *R. communis* extract containing seed storage protein (SSP, 13 kDa), ricin (65 and 70 kDa), and RCA120 (146 kDa). Prior to analysis, the molecular weight markers at 1.2 kDa and 260 kDa were added for calibration.

**Figure 4:**
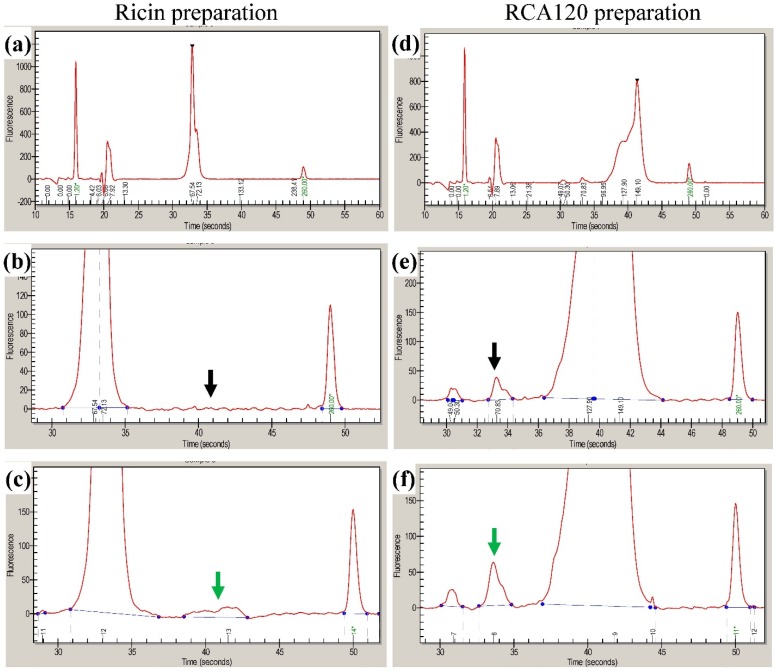
CGE analysis. Electropherogram of purified ricin (**a**–**c**) and purified RCA120 (**d**–**f**). For (**a**), the ricin preparation was diluted 1:1 and a ricin doublet peak at approximately 33 s was obtained; (**b**) expanded region around 40 s migration time where a signal of RCA120 would be expected (black arrow); (**c**) standard addition of 5% of RCA120 to the ricin preparation (0.076 mg of RCA120/mL), highlighted by a green arrow. For (**d**), the RCA120 preparation diluted 1:1 resulted in a broad peak indicating RCA120 at approximately 40 s; (**e**) expanded electropherogram of (**d**) indicating a small peak at the migration time of ricin at 34 s (black arrow). The integrated peak area was equivalent to 1.8% relative to the RCA120 peak area. (**f**) Standard addition of 2% of ricin to the RCA120 preparation (0.02 mg of ricin/mL).

Figure 3 shows a CGE electropherogram of a crude extract of *R. communis* containing *R. communis* seed storage protein (SSP), ricin, and RCA120. The molecular weight of the analytes was evaluated by the instrument software using a series of molecular weight standards analyzed in each chip and the molecular weight markers present in the sample buffer. Electropherograms of the purified ricin preparation and the purified RCA120 preparation are shown in Figure 4. In the ricin preparation the peak doublet at 65–70 kDa was attributed to ricin. No peak from RCA120—expected at 146 kDa and a running time of around 40 s—was observed (Figure 4a,b). To evaluate and confirm the CGE results, standard addition experiments were conducted. An amount of RCA120 corresponding to 5% of the concentration of ricin was added to the purified ricin preparation. In Figure 4c, a peak near the detection limit was observed (marked with a green arrow), which indicates that the RCA120 content in the purified ricin sample was less than 5%.

In the RCA120 preparation, a peak from RCA120 was obtained at 120–150 kDa and a minor peak at the migration time of ricin of around 33 s could be observed (black arrow). The concentration of ricin relative to the RCA120 in this preparation was estimated by the software to be 1.8% (Figure 4d,e). The result was confirmed by the addition of 2% of ricin relative to the RCA120 concentration. The peak attributed to ricin increased in size by a factor of approximately two (Figure 4f, green arrow).

#### 2.1.2. Molecular Weight by LC-ESI MS

**Figure 5:**
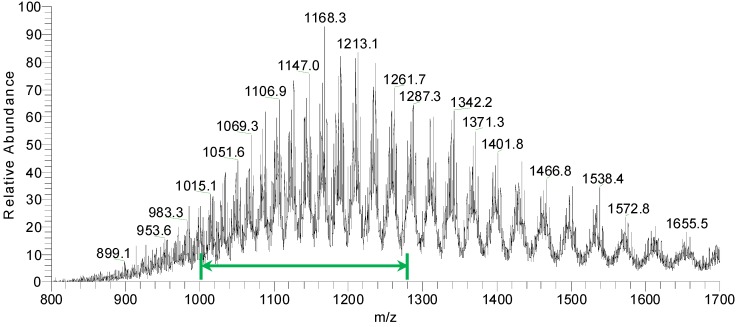
LC-ESI MS spectrum of purified ricin preparation. The highlighted range 1000–1280 *m*/*z* indicated by a green arrow was used for manual molecular weight determination.

The molecular weight of the purified, intact ricin preparation was determined based on LC-ESI MS. The molecular weight was both calculated manually from the data as well as determined from deconvoluted mass spectra. The spectra obtained showed the heterogeneity in ricin due to a varying level of glycosylation (Figure 5). Based on previous experiments and literature data, the glycosylation pattern of the mass spectrum is indicative of the cultivar used for the isolation of ricin [27].

**Figure 6:**
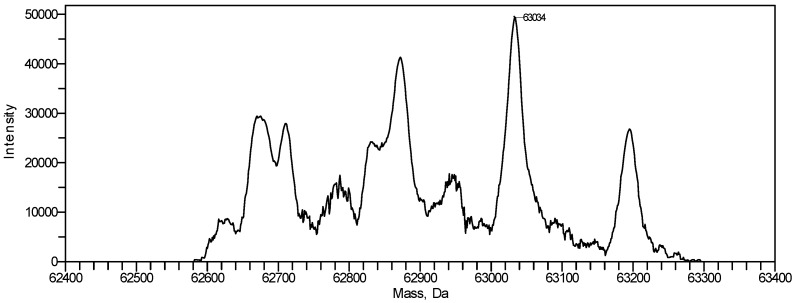
Deconvoluted mass spectrum of purified ricin preparation.

Adjacent peaks in the deconvoluted mass spectrum of the purified ricin in Figure 6 are separated by 162 mass units which correspond to different ricin glycoforms separated by the number of hexose units in the attached glycan structures. The molecular weight determined by the instrument software for the major glycoform was 63,034 Da. The manually calculated molecular weight using the peaks in the range indicated in Figure 5 was determined at 63,032 ± 2 Da. This matches well the molecular weight of previously prepared ricin from the same cultivar (unpublished) and published information [27,53,54,55].

### 2.2. Identification of Ricin and RCA120 and Analysis of Potential Trace Contaminants

#### 2.2.1. Identification of Ricin and RCA120 by Tryptic Fingerprinting and Sequencing

**Figure 7:**
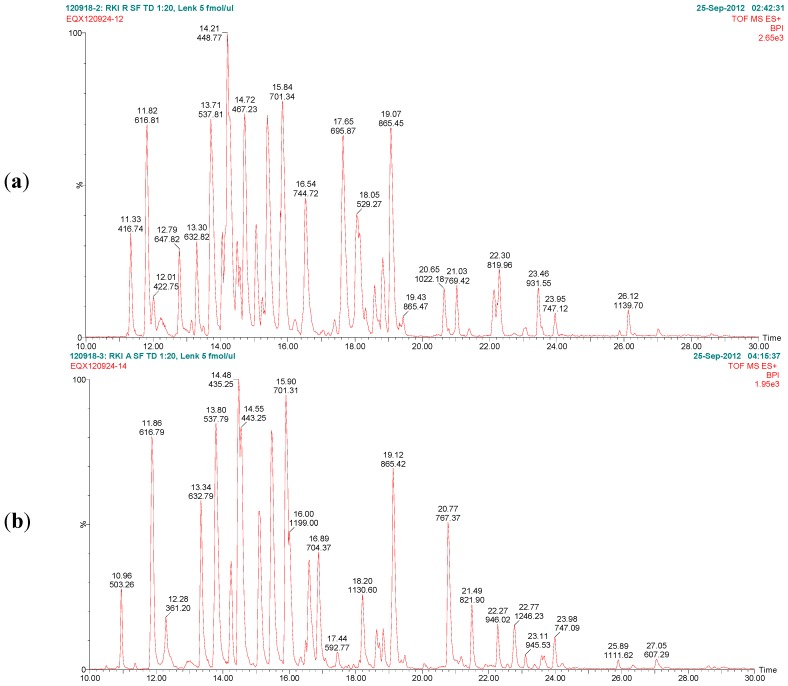
Base peak chromatograms of the trypsin digests of (**a**) purified ricin and (**b**) purified RCA120. The relative intensity is plotted against the retention time. Peaks are labeled with retention time and the *m/z* value of the base peak.

The aim of the LC-ESI MS/MS analysis (this section) and the MALDI-TOF MS/MS analysis (Section 2.2.2) was to unambiguously identify ricin and RCA120 in the protein preparations prepared from *R. communis* and to thoroughly check for potential cross-contaminations with other *R. communis* proteins. To this end, both preparations were reduced, alkylated, and underwent tryptic digestion. The tryptic fragments were analyzed and sequenced by LC-ESI MS/MS, focusing on identification of the isoforms of ricin D, ricin E, and their corresponding A and B chains, as well as RCA120. Figure 7 exemplarily shows the base peak chromatograms of the trypsin digests of ricin and RCA120 preparations obtained by LC-ESI MS/MS analysis. The peptides from ricin D observed in the ricin preparation covered 95% of the sequence of the A chain and 74% of the B chain. In the RCA120 preparation, peptides representing 90% of the A chain and 79% of the B chain of RCA120 were detected. The full sequences of ricin D, ricin E, and RCA120 are displayed in Table 1. Sequences displayed in red show peptides which were experimentally detected by combining LC-ESI MS/MS and MALDI-TOF MS/MS data (this Section and Section 2.2.2).

Table 2 and Table 3 list the theoretical trypsin digest peptides obtained from chains A and B of ricin D (Uniprot [56] ID: P02879), and those from chain B of ricin E (NCBI Genpept [57] ID: GI:225419 ) are found in Table 4.

The corresponding trypsin digest peptides from RCA120 (Uniprot [56] ID: P06750) and for chain B (NCBI Genpept [57] ID: GI:225114) are shown in Table 5 and Table 6. In each of the tables, the molecular ions of the experimentally observed peptides are indicated by bold typeface. Also, the peptides that can be used to distinguish ricin D from ricin E and RCA120 from ricin D are indicated in bold typeface.

**Table 1:** The amino acid sequences of ricin D, ricin E, and RCA120 identified in purified ricin and purified RCA120. The experimentally detected sequences using liquid chromatography-electrospray ionization-tandem mass spectrometry (LC-ESI MS/MS) and matrix-assisted laser desorption/ionization-time of flight tandem mass spectrometry (MALDI-TOF MS/MS) as trypsin digest peptides are indicated in red.

Sequence coverage of ricin preparation:	
**Ricin D chain A (Uniprot P02879, NCBI GI:132567)**	
1	IFPKQYPIIN FTTAGATVQS YTNFIRAVRG RLTTGADVRH EIPVLPNRVG LPINQRFILV
61	ELSNHAELSV TLALDVTNAY VVGYRAGNSA YFFHPDNQED AEAITHLFTD VQNRYTFAFG
121	GNYDRLEQLA GNLRENIELG NGPLEEAISA LYYYSTGGTQ LPTLARSFII CIQMISEAAR
181	FQYIEGEMRT RIRYNRRSAP DPSVITLENS WGRLSTAIQE SNQGAFASPI QLQRRNGSKF
241	SVYDVSILIP IIALMVYRCA PPPSSQF
**Ricin D chain B (Uniprot P02879, NCBI GI:132567)**	
1	ADVCMDPEPI VRIVGRNGLC VDVRDGRFHN GNAIQLWPCK SNTDANQLWT LKRDNTIRSN
61	GKCLTTYGYS PGVYVMIYDC NTAATDATRW QIWDNGTIIN PRSSLVLAAT SGNSGTTLTV
121	QTNIYAVSQG WLPTNNTQPF VTTIVGLYGL CLQANSGQVW IEDCSSEKAE QQWALYADGS
181	IRPQQNRDNC LTSDSNIRET VVKILSCGP ASSGQRWMFKN DGTILNLYSG LVLDVRASDP
241	SLKQIILYPL HGDPNQIWLP LF
**Ricin E chain B (NCBI GI:225419)**	
1	ADVCMDPEPI VRIVGRNGLC VDVRDGRFHN GNAIQLWPCK SNTDANQLWT LKRDNTIRSN
61	GKCLTTYGYP SGVYVMIYDC NTAATDATRW QIWDNGTIIN PRSSLVLAAT SGNSGTTLTV
121	QTNIYAVSQG WLPTNNTQPF VTTIVGLYGM CLQANSGKVW LEDCTSEKAE QQWALYADGS
181	IRPQQNRDNC LTTDANIKGT VVKILSCGPA SSGQRWMFKN DGTILNLYNG LVLDVRRSDP
241	SLKQIIVHPV HGNLNQIWLP LF
**Sequence coverage of RCA120 preparation:**	
**RCA120 chain A (Uniprot P06750, NCBI GI:113504)**	
1	IFPKQYPIIN FTTADATVES YTNFIRAVRS HLTTGADVRH EIPVLPNRVG LPISQRFILV
61	ELSNHAELSV TLALDVTNAY VVGCRAGNSA YFFHPDNQED AEAITHLFTD VQNSFTFAFG
121	GNYDRLEQLG GLRENIELGT GPLEDAISAL YYYSTCGTQI PTLARSFMVC IQMISEAARF
181	QYIEGEMRTR IRYNRRSAPD PSVITLENSW GRLSTAIQES NQGAFASPIQ LQRRNGSKFN
241	VYDVSILIPI IALMVYRCAP PPSSQF
**RCA120 chain B (NCBI GI:225114)**	
1	ADVCMDPEPI VRIVGRNGLC VDVFGEEFTD GNPIQLWPCK SNTDWNQLWT LRKDSTIRSD
61	GKCLTISKSS PGQQVVIYNC STATVGATRW QIWDNRTIIN PTSGLVLAAT SGNSGTKLTV
121	QTNIYAVSQG WLPTNNTQPF VTTIVGLYGM CLQANSGKVW LEDCTSEKAE QQWALYADGS
181	IRPQQNRDNC LTTDANIKGT VVKILSCGPA SSGQRWMFKN DGTILNLYNG LVLDVRRSDP
241	SLKQIIVHPV HGNLNQIWLP LF

**Table 2:** Ricin D and E chain A trypsin digest peptides (Uniprot [56] ID: P02879, NCBI Genpept [57] ID: GI:132567), reduced and carbamidomethylated. Ricin/RCA120-distinguishing peptides and observed molecular ions are given in bold.

T# ^(a)^	Amino Acid Sequence	(M+H)^+^	(M+2H)^2+^	(M+3H)^3+^	(M+4H)^4+^
T1a	IFPK	504.3180	-	-	-
**T2a-glyc**	**QYPIINFTTAGATVQSYTNFIR**	3675.6946 ^*^	1838.3509 ^*^	1225.9031 ^*^	919.6791 ^*^
T3a	AVR	345.2245	-	-	-
T4a	GR	232.1404	-	-	-
**T5a**	**LTTGADVR**	832.4523	**416.7298**	-	-
T6a	HEIPVLPNR	1074.6054	**537.8063**	-	-
**T7a**	**VGLPINQR**	896.5312	**448.7692**	-	-
**T8a**	**FILVELSNHAELSVTLALDVTNAYVVGYR**	3206.7095	1603.8584	**1069.5747**	802.4328
**T9a**	**AGNSAYFFHPDNQEDAEAITHLFTDVQNR**	3307.5038	1654.2555	1103.1728	**827.6314**
**T10a**	**YTFAFGGNYDR**	1310.5800	**655.7936**	-	-
**T11a**	**LEQLAGNLR**	1013.5738	**507.2905**	-	-
**T12a**	**ENIELGNGPLEEAISALYYYSTGGTQLPTLAR**	3440.7219	1720.8646	**1147.5788**	860.9359
**T13a**	**SFIICIQMISEAAR**	1638.8342	**819.9207**	-	-
T14a	FQYIEGEMR	1172.5404	**586.7738**	-	-
T15a	TR	276.1666	-	-	-
T16a	IR	288.2030	-	-	-
T17a	YNR	452.2252	-	-	-
T18a	R	175.1189	-	-	-
T19a	SAPDPSVITLENSWGR	1728.8551	**864.9312**	-	-
T20a	LSTAIQESNQGAFASPIQLQR	2259.1727	**1130.0900**	**753.7291**	-
T21a	R	175.1189	-	-	-
T22a	NGSK	405.2092	-	-	-
**T23a**	**FSVYDVSILIPIIALMVYR**	2212.2450	**1106.6261**	**738.0865**	-
T24a	CAPPPSSQF	990.4349	**495.7211**	-	-

^(a)^ Trypsin digest peptides numbered from the amino terminal of the polypeptide chain. * Main glycopeptide.

**Table 3:** Ricin D chain B trypsin digest peptides (Uniprot [56] ID: P02879, NCBI Genpept [57] ID: GI:132567), reduced and carbamidomethylated. Ricin/RCA120 distinguishing peptides and observed molecular ions are given in bold.

T# ^(a)^	Amino Acid Sequence	(M+H)^+^	(M+2H)^2+^	(M+3H)^3+^	(M+4H)^4+^
T1b	ADVCMDPEPIVR	1401.6501	**701.3287**	-	-
T2b	IVGR	444.2929	-	-	-
**T3b**	**NGLCVDVR**	932.4618	**466.7345**	-	-
T4b	DGR	347.1673	-	-	-
**T5b**	**FHNGNAIQLWPCK**	1584.7740	**792.8906**	-	-
**T6b**	**SNTDANQLWTLK**	1390.6961	**695.8517**	-	-
T7b	R	175.1189	-	-	-
**T8b**	**DNTIR**	**618.3205**	-	-	-
T9b	SNGK	405.2092	-	-	-
**T10b**	**CLTTYGYSPGVYVMIYDCNTAATDATR**	3063.3532	1532.1802	**1021.7893**	766.5938
**T11b-glyc**	**WQIWDNGTIINPR**	2991.2987 ^*^	1496.1530 ^*^	997.7711 ^*^	748.5801 ^*^
**T12b-glyc**	**SSLVLAATSGNSGTTLTVQTNIYAVSQGWLPTNNTQPFVTTIVGLYGLCLQANSGQVWIEDCSSEK**	[7047.4666]	-	-	-
T13b	AEQQWALYADGSIRPQQNR	2231.0952	**1116.0512**	**744.3699**	-
**T14b**	**DNCLTSDSNIR**	1294.5692	**647.7882**	-	-
**T15b**	**ETVVK**	575.3399	-	-	-
T16b	ILSCGPASSGQR	1232.6052	**616.8062**	-	-
T17b	WMFK	**611.3010**	**306.1541**	-	-
**T18b**	**NDGTILNLYSGLVLDVR**	1862.0018	**931.5045**	-	-
**T19b**	**ASDPSLK**	717.3777	359.1925	-	-
**T20b**	**QIILYPLHGDPNQIWLPLF**	2277.2430	**1139.1251**	**759.7525**	-

^(a)^ Trypsin digest peptides numbered from the amino terminal of the polypeptide chain. * Main glycopeptide.

**Table 4:** Ricin E chain B trypsin digest peptides (NCBI Genpept [57] ID: GI:225419), reduced and carbamidomethylated. Ricin D/E-distinguishing sequence and observed molecular ions are given in bold.

T# ^(a)^	Amino Acid Sequence	(M+H)^+^	(M+2H)^2+^	(M+3H)^3+^	(M+4H)^4+^
T1b	ADVCMDPEPIVR	1401.6501	**701.3287**	-	-
T2b	IVGR	444.2929	-	-	-
T3b	NGLCVDVR	932.4618	**466.7345**	-	-
T4b	DGR	347.1673	-	-	-
T5b	FHNGNAIQLWPCK	1584.7740	**792.8906**	-	-
T6b	SNTDANQLWTLK	1390.6961	**695.8517**	-	-
T7b	R	175.1189	-	-	-
T8b	DNTIR	618.3205	-	-	-
T9b	SNGK	405.2092	-	-	-
T10b	CLTTYGYPSGVYVMIYDCNTAATDATR	3063.3532	1532.1802	**1021.7893**	766.5938
T11b glyc	WQIWDNGTIINPR	-	1496.1530 ^*^	997.7711 ^*^	748.5801 ^*^
**T12b glyc**	**SSLVLAATSGNSGTTLTVQTNIYAVSQGWLPTNNTQPFVTTIVGLYGMCLQANSGK**	[5831.9258]	-	-	-
**T13b**	**VWLEDCTSEK**	1266.5670	**633.7871**	-	-
T14b	AEQQWALYADGSIRPQQNR	2231.0952	**1116.0512**	**744.3699**	-
**T15b**	**DNCLTTDANIK**	1264.5838	**632.7955**	-	-
T16b	**GTVVK**	503.3187	-	-	-
T17b	ILSCGPASSGQR	1232.6052	**616.8062**	-	-
T18b	WMFK	**611.3010**	**306.1541**	-	-
**T19b**	**NDGTILNLYNGLVLDVR**	1889.0127	**945.0100**	-	-
T20b	R	175.1189	-	-	-
**T21b**	**SDPSLK**	646.3406	323.6739	-	-
**T22b**	**QIIVHPVHGNLNQIWLPLF**	2238.2545	**1119.6309**	**746.7564**	-

^(a)^ Trypsin digest peptides numbered from the amino terminal of the polypeptide chain. * Main glycopeptide.

**Table 5:** RCA120 chain A (Uniprot [56] ID: P06750, NCBI Genpept [57] ID: GI:113504) trypsin digest peptides, reduced and carbamidomethylated. Ricin/RCA120-distinguishing peptides and observed molecular ions are given in bold.

T# ^(a)^	Amino Acid Sequence	(M+H)^+^	(M+2H)^2+^	(M+3H)^3+^	(M+4H)^4+^
T1a	IFPK	504.3180	-	-	-
**T2a glyc**	**QYPIINFTTADATVESYTNFIR**	3734.6841 ^*^	1867.8457 ^*^	1245.5662 ^*^	934.4265 ^*^
T3a	AVR	345.2245	-	-	-
**T4a**	**SHLTTGADVR**	1056.5432	528.7752	-	-
T5a	HEIPVLPNR	1074.6054	**537.8063**	-	-
**T6a**	**VGLPISQR**	869.5203	**435.2638**	-	-
**T7a**	**FILVELSNHAELSVTLALDVTNAYVVGCR**	3203.6768	1602.3420	**1068.5638**	801.6747
**T8a**	**AGNSAYFFHPDNQEDAEAITLFTDVQNSFTFAFGGNYDR**	4514.0020	2257.5046	1505.3389	**1129.2560**
**T9a**	**LEQLGGLR**	885.5152	**443.2612**	-	-
**T10a**	**ENIELGTGPLEDAISALYYYSTCGTQIPTLAR**	3516.7202	1758.8637	**1172.9116**	879.9355
**T11a**	**SFMVCIQMISEAAR**	1642.7750	**821.8911**	-	-
T12a	FQYIEGEMR	1172.5404	**586.7738**	-	-
T13a	TR	276.1666	-	-	-
T14a	IR	288.2030	-	-	-
T15a	YNR	452.2252	-	-	-
T16a	R	175.1189	-	-	-
T17a	SAPDPSVITLENSWGR	1728.8551	**864.9312**	-	-
T18a	LSTAIQESNQGAFASPIQLQ R	2259.1727	1130.0900	**753.7291**	-
T19a	R	175.1189	-	-	-
T20a	NGSK	405.2092	-	-	-
**T21a**	**FNVYDVSILIPIIALMVYR**	2239.2559	**1120.1316**	**747.0902**	-
T22a	CAPPPSSQF	990.4349	**495.7211**	-	-

^(a)^ Trypsin digest peptides numbered from the amino terminal of the polypeptide chain. * Main glycopeptide.

**Table 6:** RCA120 chain B (NCBI Genpept [57] ID: GI:225114) trypsin digest peptides, reduced and carbamidomethylated. Ricin/RCA120-distinguishing peptides and observed molecular ions are given in bold.

T# ^(a)^	Amino Acid Sequence	(M+H)^+^	(M+2H)^2+^	(M+3H)^3+^	(M+4H)^4+^
T1b	ADVCMDPEPIVR	1401.6501	**701.3287**	-	-
T2b	IVGR	444.2929	-	-	-
**T3b**	**NGLCVDVFGEEFTDGNPIQLWPCK**	2795.2803	1398.1438	**932.4316**	699.5755
**T4b**	**SNTDWNQLWTLR**	1533.7444	**767.3758**	-	-
T5b	K	147.1128	-	-	-
**T6b**	DSTIR	591.3096	-	-	-
T7b	SDGK	406.1932	-	-	-
**T8b**	**CLTISK**	**721.3913**	**361.1993**	-	-
**T9b glyc**	**SSPGQQVVIYNCSTATVGATR**	3366.4960 ^*^	1683.7516 ^*^	**1122.8369** ^*^	842.3795 ^*^
**T10b-glyc**	**WQIWDNR**	2395.9657 ^*^	1198.4865 ^*^	799.3268 ^*^	-
**T11b**	**TIINPTSGLVLAATSGNSGTK**	2002.0815	**1001.5444**	**668.0320**	-
**T12b glyc**	**LTVQTNIYAVSQGWLPTNNTQPFVTTIVGLYGMCLQANSGK**	[4485.2581]	-	-	-
T13b	VWLEDCTSEK	1266.5670	**633.7871**	-	
T14b	AEQQWALYADGSIRPQQNR	2231.0952	**1116.0512**	**744.3699**	**-**
T15b	DNCLTTDANIK	1264.5838	**632.7955**	**-**	**-**
T16b	GTVVK	503.3187	**-**	**-**	**-**
T17b	ILSCGPASSGQR	1232.6052	**616.8062**	**-**	**-**
T18b	WMFK	**611.3010**	**306.1541**	**-**	**-**
T19b	NDGTILNLYNGLVLDVR	1889.0127	**945.0100**	**-**	**-**
T20b	R	175.1189	**-**	**-**	**-**
T21b	SDPSLK	646.3406	323.6739	**-**	**-**
T22b	QIIVHPVHGNLNQIWLPLF	2238.2545	**1119.6309**	**746.7564**	**-**

^(a)^ Trypsin digest peptides numbered from the amino terminal of the polypeptide chain. * Main glycopeptide.

In the E isoform of ricin, the amino acid sequence of chain A is identical to that of ricin D. The differences between D and E are located at the B chain, where approximately half the chain at the carboxyl terminal end has the same amino acid sequence as RCA120. In the *R. communis* cultivar used here, both ricin isoforms were found. Several peptides detected in the purified ricin preparation were attributed to ricin E, e.g., T13b, T15b, T19b, and T22b (Table 4).

Besides the sequence GI:225419 [17], two other sequences for ricin E, GI:2169612 and GI:225896 [18], are published in the NCBI Genpept database (The National Center for Biotechnology Information, National Library of Medicine, Bethesda, MD, USA) [57]. The peptide molecular ions at 616^2+^ and 746^3+^ matched T17b and T22b of GI:225419 and distinguished it from the other ricin E sequences (GI:225896, GI:2169612).

The ricin E sequence GI:225419 has a Pro-Ser at position 70–71 [17] identical to that published for ricin D by the same group [58,59]. Pro70-Ser71 in the T10b peptide from this ricin preparation could not be confirmed (see Supplementary Information, Figure S1). Instead, data supported Ser-Pro at position 70–71, which corresponds to other sequences published for ricin E (GI:225896 [18], GI:2169612) and ricin D (GI:132567 [60]). The discrepancy could be due to an actual difference between isoforms or flaws in the older data using sequencing methods available at that time. A comparison to X-ray structures could not be performed, since no structure for the B chain of ricin E is available so far.

The NCBI database contains a number of sequence variants for the B chain of RCA120, where sequence GI:225114 differs from other RCA120 sequences, e.g. Uniprot P06750 (NCBI Genpept GI:113504; X-ray structure: 1RZO). In the purified RCA120 preparation, the two peptides identified as T9 glycopeptide and T11 of the RCA120 chain B sequence GI:225114 distinguished this isoform from other RCA120 sequences, e.g., GI:113504 (Table 6; Supplementary Information Figures S2 and S3). The product ion spectrum of the T3 peptide of chain B, however, was consistent with the amino acid sequence of GI:113504 and not GI:225114 (Supplementary Information Figure S4). Again, this inconsistency could be due to lower reliability of older sequencing methods.

Twenty-eight peptides from ricin and 27 from RCA120 were detected by the nano-LC-MS method used in this investigation. Nineteen of the ricin peptides were proteotypic, *i.e.*, not found in other proteins including RCA120. For further in-depth characterization, increased sequence coverage could be obtained on sections resistant to trypsin by using proteases with alternative cleavage specificity, e.g., chymotrypsin or pepsin.

For identification and quantification, sequence coverage is less important, and instrument capacity and sensitivity requirements might restrict the number of monitored peptides. Peptides suitable for identification and quantification of ricin, *i.e.*, proteotypic and detected at high intensity, are consistent with those proposed earlier [44,61,62] with the exception of T19b, which is polar and was not retained by the trapping column in the nano-LC system used here. Diagnostic peptides for ricin with high identification potential observed here were T5, T7, T9, T10, and T11 from the A chain and T6, T11, T18, and T20 from the B chain. When the sample was reduced and alkylated before digestion, both T13 from chain A and T14 from chain B (ricin D) also exhibited good electrospray response and excellent MS/MS sequence information. B chain peptides T3 and T5 were both prone to deamidation, which made them less suitable as target peptides for quantification and unequivocal identification.

In RCA120 T6 and T9 from chain A together with T4 from chain B were conserved in all isoforms and should be selected as primary target peptides for unequivocal identification of RCA120 as well as T11a when the sample is reduced and alkylated. Ricin E could be distinguished from ricin D by the B chain peptides highlighted in Table 4, primarily T13, T15, T19, and T22.

Peptides common to ricin and RCA120 suitable for detection and identification of both proteins when combined with proteotypic peptides were T6, T14, T19, and T20 from chain A and T13 and T16 from chain B of ricin. The disulfide linked peptide connecting the A and B chains in ricin and RCA120 had a relatively low electrospray response, but in samples at toxicologically relevant concentrations it was useful as an indication of potentially active toxin.

When matched to the NCBIdatabase, the majority of the diagnostic ricin peptides were unique, except for T5a which was found in a *Pomacea flagellata* hemagglutinin (a freshwater snail) and B chain T3 which appeared in related ribosome-inactivating proteins [44]. The glycopeptide T11b also matched *Viscum album* lectin 1 (viscumin) and T19b three other proteins. As the databases continue to grow, overlaps with both functionally related and purely coincidental new proteins could occur.

Depending on the scenario, the number of peptides required for identification and their sequence coverage need to be considered. In contrast to recommendations for proteomic identification of unknown proteins in complex mixtures in the life sciences [63,64,65], the identification of target proteins in public health and forensic investigations involves the comparison of experimental data with authentic reference standards. Criteria for chromatographic retention time and ion ratios as well as the mass of digestion products and MS/MS sequence information will need to be specified [66].

The World Anti-Doping Agency (WADA) has published detailed identification criteria for proteins. Reporting of a unique amino acid sequence and minimum sequence coverage of 10% was specified [67]. Identification criteria for ricin in the context of verification of the Chemical Weapons Convention was proposed by a working group under the OPCW Scientific Advisory Board [68]. The MS criteria recommended reporting a minimum of two peptides from each of the chains with detailed experimental data and, furthermore, that the uniqueness of the peptides needs to be specified. Supporting data from immunological techniques, ricin functionality assay, molecular weight determination or PCR was additionally required for unambiguous identification.

Generally, no peptides from other *R. communis* components were detected in the trypsin digests of purified ricin and purified RCA120, e.g., 2S-albumin or the peptide biomarkers RCB-1 to -3 [69]. The next step was to analyze potential cross-contaminations of RCA120 in the purified ricin preparation and, *vice versa*, ricin in the purified RCA120 preparation. The concentration of the impurities in the preparations was calculated using the protein concentrations obtained from UV_280_ measurement and the responses of peptides specific for ricin and RCA120.

The amount of RCA120 in the purified ricin sample was calculated from the peak areas determined from the extracted ion chromatograms, using the RCA120 preparation as the analytical standard. Similarly, the amount of ricin in purified RCA120 was determined using the ricin preparation as the analytical standard. Based on this procedure, the amount of RCA120 in the ricin preparation was 1.4% ± 0.4% relative to ricin based on the average of chain A peptides, and 4.5% ± 1.5% based on chain B peptides. Therefore, the purity of ricin was specified to **≥**97% based on the average of chain A and B peptides.

For the *R. communis* agglutinin preparation, the amount of ricin found was 0.5% ± 0.2% calculated for chain A peptides and 1.4% ± 0.7% for chain B peptides, resulting in an average purity of >99%. For both protein preparations, these results were in good agreement with the purity estimated from CGE analysis.

Overall, the purity of the ricin and the RCA120 preparation characterized in this work was very good. While for a commercial preparation of ricin a similar purity of 96% was reported, the corresponding RCA120 preparation showed a purity of only 68% [43]. Additionally, our toxin preparations were derived from a defined *R. communis* cultivar, while the cultivar used for purification concerning the commercial products is not known.

As described above, the concentration of RCA120 in the purified ricin sample was consistently higher when calculated using chain B peptides than peptides from chain A. The same observation was made for ricin in the purified RCA120 sample. This was unexpected, considering the nature of the sample and the purification scheme used. The purification process consisted of an acid extraction at pH 4 and ammonium sulfate precipitation of proteins. Affinity separation using a lactosyl-Sepharose-4B column was performed to isolate ricin and RCA120 from other constituents in the precipitate. Finally, size exclusion chromatography was used to separate ricin from RCA120. A possible explanation for the excess of chain B peptides over chain A peptides measured as contamination in both preparations could be a decomposition process or a reduction of the A–B interchain disulfide bond during the extraction and precipitation process. Chain B of both ricin and RCA120 would be isolated together with the intact proteins in the affinity step and could end up in the ricin size exclusion chromatography fraction to an extent depending on the size exclusion separation efficiency.

The amount of ricin E was determined in the ricin preparation using the chain B peptides that distinguish the E isoform from ricin D (Table 4). However, since they are shared with RCA120, the result had to be corrected by the content of RCA120 which can be determined from the intensity of the relevant RCA120 peptides, e.g., T6, T9, and T11 from chain A and T3 and T4 from chain B. Based on this procedure, the ratio of ricin D to ricin E was estimated to be close to one for the ricin preparation purified from *R. communis carmencita* in this work (data not shown).

#### 2.2.2. Analysis of Trace Contaminants by MALDI-TOF MS/MS Analysis

The aim of the following experiments was to identify any impurities which might be present in both the ricin and the RCA120 preparation. As described in Section 2.1.1, the separation of larger amounts of both purified proteins by SDS-PAGE resulted in a number of faint lower-molecular-weight protein bands of unknown identity (Figure 2). It has remained unclear from the experiments conducted so far whether these protein bands were fragmented ricin or RCA120 proteins or irrelevant contaminants co-purified from the seeds. Therefore, both purified preparations were separated on an SDS-PAGE, and Coomassie-stained bands were cut out, and proteins were reduced, alkylated, and digested with trypsin, followed by a MALDI-TOF MS/MS analysis.

**Figure 8:**
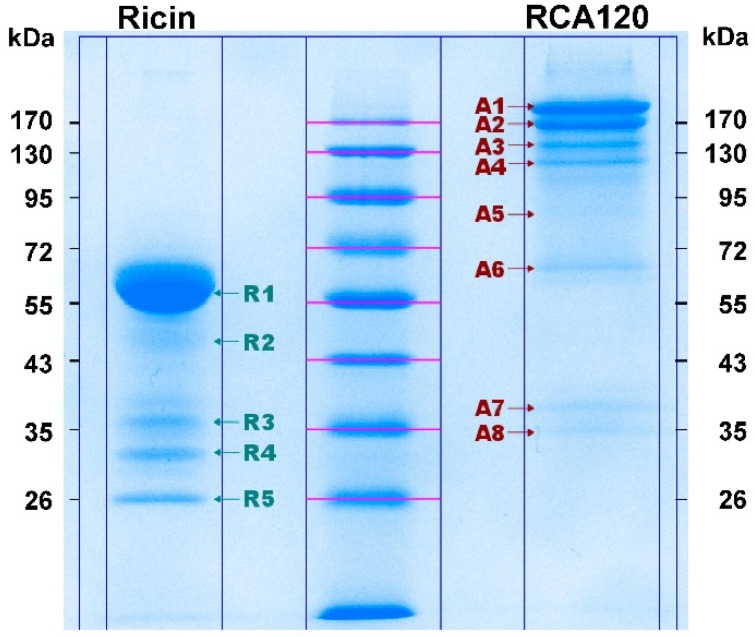
Separation of purified ricin and RCA120 by gel electrophoresis for subsequent MALDI-TOF MS/MS analysis. Purified ricin (15.1 µg, left) or purified RCA120 (10.3 µg, right) were separated on a 10% SDS-PAGE. The indicated protein bands were cut out for further analysis: bands R1 through R5 for ricin and bands A1 through A8 for RCA120, respectively. For an in-gel digest, bands were cut out, reduced, and alkylated, used for tryptic digestion, and finally eluted from the gel, followed by MALDI-TOF MS/MS analysis.

Figure 8 shows the Coomassie-stained gel which was used to cut out protein bands. The gel pieces corresponding to the individual protein bands were numbered sequentially R1 through R5 for the purified ricin preparation and A1 through A8 for the purified RCA120 preparation.

**Figure 9:**
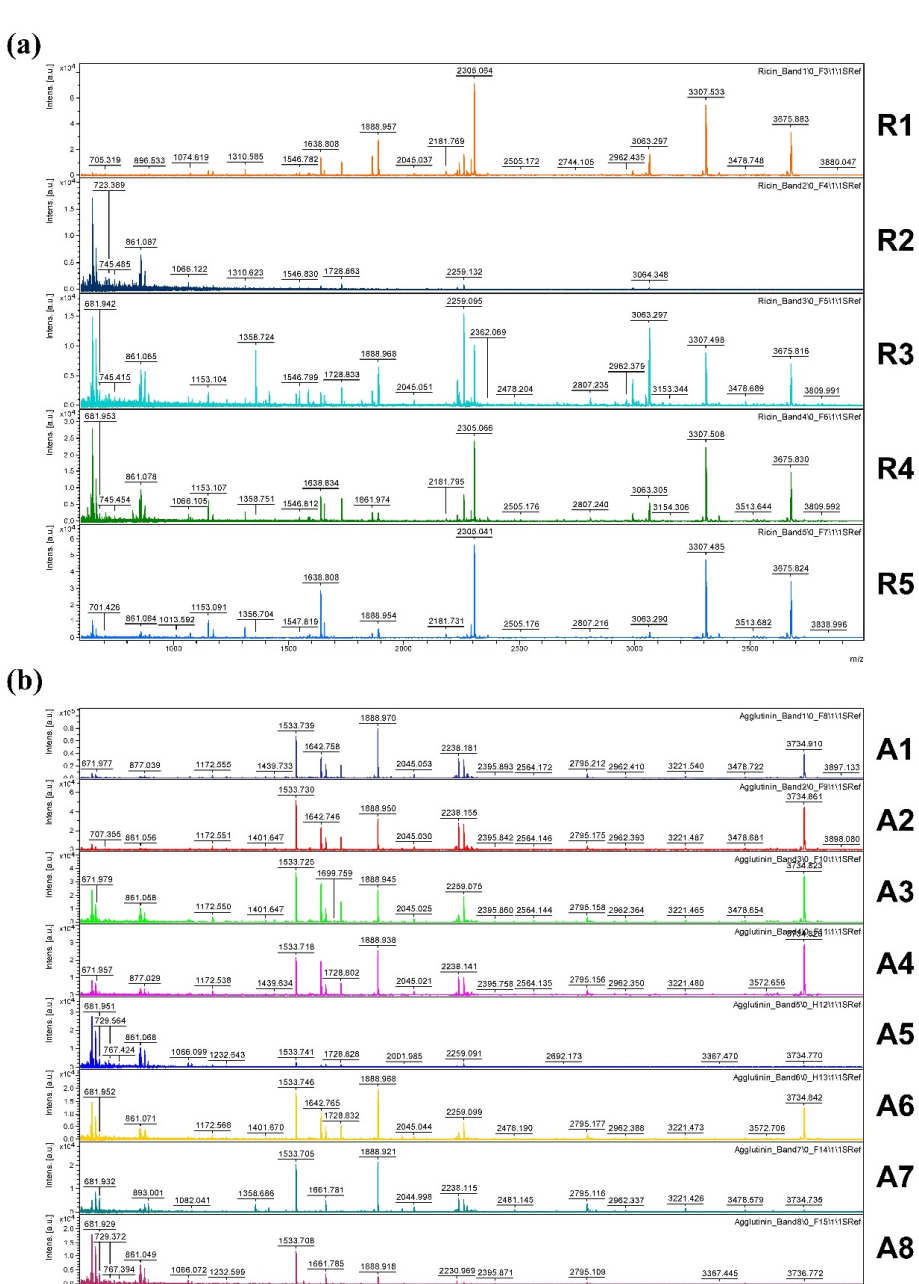
Overview spectra of trypsin-digested samples for gel lanes containing the purified ricin or RCA120 preparation. From top to bottom: analysis of (**a**) bands R1 through R5 of the ricin preparation and (**b**) bands A1 through A8 of the RCA120 preparation. Peaks are labeled with the corresponding *m*/*z* value of the base peak.

Figure 9 shows an overview of resulting mass spectra of trypsin-digested ricin and RCA120 bands. All ricin bands showed similar mass spectral patterns, except for band 2, presumably because of a lower protein amount. For the purified RCA120 preparation, all mass spectra were similar, except for bands 5 and 8, again presumably because of low protein amounts analyzed.

Table 7, Table 8 and Table 9 list the specific peptides identified as products of trypsin digestion from chains A and B of ricin D (Uniprot [56] ID: P02879) as well from chain B of ricin E (Uniprot [56] ID: Q41143) which could be found in the mass spectra of the indicated gel bands.

**Table 7:** Ricin D chain A-specific peptides obtained by trypsin digestion (Uniprot [56] ID: P02879, NCBI Genpept [57] ID: GI:132567) with one missed cleavage, reduced and carbamidomethylated in the mass range of 600–4000, and obtained by mass spectrometry analysis of the respective gel bands.

Position	Amino Acid Sequence	(M+H)^+^	R1 *	R2 *	R3 *	R4 *	R5 *
67–74	VGLPINQR	896.5312	x	-	-	x	x
161–169	LEQLAGNLR	1013.5738	x	x	-	x	x
150–160	YTFAFGGNYDR	1310.5800	x	x	x	x	x
190–203	SFIICIQMISEAAR	1638.8342	x	x	x	x	x
67–83	LTTGADVRHEIPVLPNR	1888.0399	x	-	x	x	x
150–169	YTFAFGGNYDRLEQLAGNLR	2305.1360	x	-	x	x	x
121–149	AGNSAYFFHPDNQEDAEAIT HLFTDVQNR	3307.5038	x	-	x	x	x

* R1–R5: Ricin band number of the one-dimensional gel electrophoresis; please compare Figure 8. Indicated by “x” is the presence of the respective trypsin digest peptide shown.

**Table 8:** Ricin D chain B-specific peptides obtained by trypsin digestion (Uniprot [56] ID: P02879, NCBI GenPept [57] ID: GI:132567) with one missed cleavage, reduced and carbamidomethylated in the mass range of 600–4000, and obtained by mass spectrometry analysis of the respective gel bands.

Position	Amino Acid Sequence	(M+H)^+^	R1 *	R2 *	R3 *	R4 *	R5 *
355–366	SNTDANQLWTLK	1390.6961	x	-	x	-	-
355–367	SNTDANQLWTLKR	1546.7972	x	x	x	x	x
330–342	FHNGNAIQLWPCK	1584.7740	x	-	x	x	-
534–550	NDGTILNLYSGLVLDVR	1862.0018	x	-	x	x	x
365–391	CLTTYGYSPGVYVMIYDCNTAATDATR	3063.3532	x	x	x	x	x

* R1–R5: Ricin band number of the one-dimensional gel electrophoresis; please compare Figure 8. Indicated by “x” is the presence of the respective trypsin digest peptide shown.

**Table 9:** Ricin E chain B-specific peptides obtained by trypsin digestion (Uniprot [56] ID: Q41143) with one missed cleavage, reduced and carbamidomethylated in the mass range of 600–4000, and obtained by mass spectrometry analysis of the respective gel bands.

Position	Amino Acid Sequence	(M+H)^+^	R1 *	R2 *	R3 *	R4 *	R5 *
220–236	NDGTILNLYNGLVLDVR	1889.0127	x	-	x	x	x

* R1–R5: Ricin band number of the one-dimensional gel electrophoresis; please compare Figure 8. Indicated by “x” is the presence of the respective trypsin digest peptide shown.

All ricin bands R1 to R5 contained ricin chains A and B. Ricin E chain B could be observed in the ricin bands 1, and 3 to 5. Cross-contamination of RCA120 in the ricin preparation was found in bands R1 and R3, but only in one RCA120 peptide fragment (*m*/*z* 1533, SNTDWNQLWTLR). Based on these data, cross-contamination of RCA120 in the ricin preparation was very low, corroborating results obtained by the LC-ESI MS/MS analysis.

The corresponding specific trypsin digest peptides from RCA120, Uniprot [56] ID: P06750, are displayed in Table 10 and Table 11. In the RCA120 preparation, only RCA120 peptides (Uniprot [56] ID: P06750) and no ricin peptides were detected by MALDI-TOF MS analysis. Measured peptides belonged to RCA120 chains A and B, while band 8 showed peptides for RCA120 chain B only.

In summary, one-dimensional gel electrophoresis showed that the ricin preparation can be separated into five distinct bands with one main fragment at about 63 kDa, whereas purified RCA120 can be separated into eight bands with two main fragments running at around 170 kDa. The identity of the ricin and RCA120 bands was verified by MALDI-TOF-MS/MS analysis after trypsin digestion. All ricin bands contained ricin chains A and B of ricin D (Uniprot [56] ID: P02879). Ricin bands 1 and 3 to 5 also contained ricin E (Uniprot [56] ID: Q41143), while bands R1 and R3 also contained one peptide fragment (*m*/*z* 1533, SNTDWNQLWTLR) of RCA120. The cross-contamination was negligible. In purified RCA120 only RCA120 (Uniprot [56] ID: P06750) and no ricin was detected. Measured peptides belonged to RCA120 chains A and B, while band 8 only showed peptides for RCA120 chain B. Most importantly, the MALDI-TOF-MS/MS experiments gave no indication of the presence of irrelevant proteins co-purified from the *R. communis* seeds.

**Table 10:** RCA120 chain A-specific peptides obtained by trypsin digestion (Uniprot [56] ID: P06750, NCBI Genpept [57] ID: GI113504) with one missed cleavage, reduced and carbamidomethylated in the mass range of 600–4000, and obtained by mass spectrometry analysis of the respective gel bands.

Position	Amino Acid Sequence	(M+H)^+^	A1 *	A2 *	A3 *	A4 *	A5 *	A6 *	A7 *	A8 *
73–80	VGLPISQR	869.5203	x	x	-	-	-	-	-	-
150–157	LEQLGGLR	885.5152	x	x	x	x	-	x	-	-
190–203	SFMVCIQMISEAAR	1642.7750	x	x	x	x	x	x	-	-
64–80	HEIPVLPNRVGLPISQR	1925.1079	x	x	-	-	-	-	-	-
263–281	FNVYDVSILIPIIALMVYR	2238.2545	x	x	x	x	-	x	x	-
158–189	ENIELGTGPLEDAISALYYYSTCGTQIPTLAR	3516.7202	x	x	x	x	-	x	-	-

* A1–A8: RCA120 band number of the one-dimensional gel electrophoresis; please compare Figure 8. Indicated by “x” is the presence of the respective trypsin digest peptide shown.

**Table 11:** RCA120 chain B-specific peptides obtained by trypsin digestion (Uniprot [56] ID: P06750, NCBI Genpept [57] ID: GI225114) with one missed cleavage, reduced and carbamidomethylated in the mass range of 600–4000, and obtained by mass spectrometry analysis of the respective gel bands.

Position	Amino Acid Sequence	(M+H)^+^	A1 *	A2 *	A3 *	A4 *	A5 *	A6 *	A7 *	A8 *
343–354	SNTDWNQLWTLR	1533.7444	x	x	x	x	x	x	x	x
343–355	SNTDWNQLWTLRK	1661.8394	x	x	x	x	x	x	x	x
319–342	NGLCVDVFGEEFTDGNPIQLWPCK	2795.2803	x	x	x	x	-	x	x	x

* A1–A8: RCA120 band number of the one-dimensional gel electrophoresis; please compare Figure 8. Indicated by “x” is the presence of the respective trypsin digest peptide shown.

### 2.3. Detection of Ricin and RCA120 Using Antibody-Based Techniques

In a next step, both the ricin and the RCA120 preparation purified in this work were detected using immunological methods. Two different sandwich enzyme-linked immunosorbent assays (ELISA) were used, one preferentially detecting ricin with only little cross-reactivity with RCA120 and the other one preferentially detecting RCA120 with low cross-reactivity with ricin. The ricin-specific ELISA was based on a combination of two monoclonal antibodies and detected both chains of ricin (R109 and R18 [70], Table 12), while the RCA120-specific ELISA combined a monoclonal with a polyclonal chicken antibody (mAb ARK4 [71] and chicken IgY RC22 [72], Table 12).

**Table 12:** Monoclonal and polyclonal antibodies used for immunological detection.

Name	Isotype	Specificity Indirect ELISA				Sandwich ELISA	
		Ricin	RCA120	A Chain	B Chain	Ricin	RCA120
R18	mAb, IgG1κ	+++	+	+++	-	+++	+
R109	mAb, IgG1κ	+++	++++	-	+++	++++	+
ARK4 ^§^	mAb, IgG	+	++++	n.d.	n.d.	+	+++
RC22	pAb, IgY	++++	++++	++++	++++	+++	+++

^§^ mAb ARK4 kindly provided by M. Avondet, Spiez Laboratory, Switzerland; reactivity of the indicated antibodies to ricin, RCA120, ricin A chain or ricin B chain is qualitatively indicated: - no reaction, + weak reaction, +++ strong reaction, ++++ very strong reaction; n.d., not determined.

In a validation study for the ricin-specific ELISA, the half maximal effective concentration (EC_50_) of the ELISA as the point of highest precision with respect to quantification was determined at 115 ± 32 pg/mL with a limit of detection of 2 pg/mL. The working range of the ricin-specific ELISA as the range in which obtained results have a precision of <20% and a trueness of 80%–120% was experimentally determined, and the lower and upper limits of quantification were determined at 5 pg/mL and 708 pg/mL, respectively. Intra-assay and inter-assay coefficients of variation were determined at 6% and 13% at the EC_50_ value, respectively, with *n* = 10 as the number of intra- or inter-assay repetitions performed in duplicate (data not shown).

**Figure 10:**
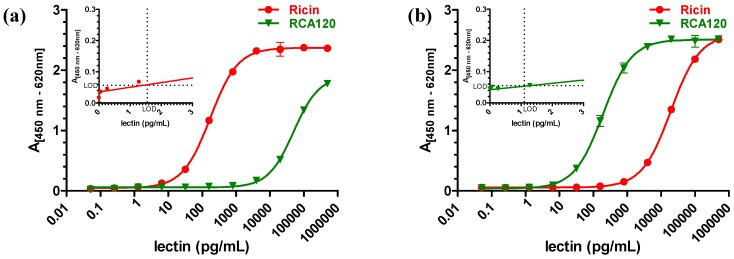
Sandwich ELISA for detection of purified ricin and purified RCA120. (**a**) A sandwich ELISA preferentially detecting ricin based on mAb R109 as capture antibody and biotinylated mAb R18 as detection antibody was used to measure serial dilutions of the purified ricin (red) or purified RCA120 (green) preparation; (**b**) A sandwich ELISA preferentially detecting RCA120 based on mAb ARK4 as capture reagent and biotinylated pAb RC22 as detection reagent was used to measure serial dilutions of ricin (red) or RCA120 (green). Both ELISAs were amplified using a polyHRP-conjugate and TMB substrate. Absorption was measured at 450 nm with a reference wavelength of 620 nm. The absorption was plotted against the logarithmic concentrations of ricin or RCA120, respectively. Results displayed are based on two independent experiments performed in duplicate each. Inset: enlargement showing the low concentration range (linear) and estimated limit of detection (LOD = mean of blank + 3.29 × SD).

Using this ELISA, purified ricin could be detected down to around 3 pg/mL (Figure 10a). In order to test for specificity, the related RCA120 was analyzed using the same sandwich ELISA. As shown in Figure 10a, when equivalent concentrations of ricin and RCA120 were tested in parallel, ricin was preferentially detected (300-fold difference at the EC_50_). However, at concentrations above 4 ng/mL, RCA120 was detected as well. No cross-reactivity except for RCA120 to a broad range of other lectins and toxins was detected (data not shown). Still, based on the observed reactivity to both analytes, in a mixture containing ricin along with RCA120, assuming that both proteins were present in significant amounts, the ELISA would not be able to distinguish both molecules reliably. An alternative interpretation of the data would be that the ricin-specific ELISA is specific for ricin only and detects the minute contamination of ricin within the RCA120 preparation (below 1% according to 2.2.1); this seems unlikely on the basis of the high sequence homology between both analytes, but cannot be ruled out since the precise epitopes recognized by both mAbs are not known. With respect to sensitivity, this ELISA is among the most sensitive sandwich ELISAs published to date. Various ELISAs have been developed by different groups [70,73,74,75,76,77,78,79,80,81,82,83]. Only a few of them are able to quantify ricin with detection limits down to a few pg/mL with limits of detection of 10 fg/mL [84], 2 pg/mL [70], 8 pg/mL [85], and 40 pg/mL [86]. Several ricin-specific ELISAs developed in different laboratories have been evaluated in a common ricin proficiency test panel organized in the framework of the EQuATox project, and results are presented in different manuscripts in this special issue of *Toxins* [42,87].

Using the combination of mAb ARK4 with pAb RC22, purified RCA120 could be detected with a limit of detection around 1 pg/mL (Figure 10b). The EC_50_ was determined at 117 pg/mL ± 27 pg/mL. The working range of the agglutinin-specific ELISA (precision of <20%; trueness of 80%–120%) was experimentally determined and the lower and upper limits of quantification were determined at 3 pg/mL and 1549 pg/mL, respectively. Intra-assay and inter-assay coefficients of variation were determined at 4% and 6% at the EC_50_ value, respectively, with *n* = 10 as number of intra- or inter-assay repetitions performed in duplicate (data not shown).

When equivalent concentrations of RCA120 and ricin were tested in parallel with this RCA120-specific ELISA, RCA120 was preferentially detected (100-fold difference at the EC_50_). Again, at concentrations above 1 ng/mL, ricin was detected as well (Figure 10b). Similar to the above, in a mixture containing significant amounts of ricin along with RCA120, the ELISA would not be able to distinguish both molecules reliably.

With respect to the ricin PT organized in the framework of the EQuATox project, both ELISAs were used to quantify the sample materials offered after spiking the purified ricin or RCA120 preparations into different liquid matrices (see this issue of *Toxins*: [42]).

### 2.4. Functional Activity of Ricin and RCA120

Ricin is a lectin and functions as an enzyme within the body: after binding to distinct carbohydrate structures on the cell surface via its B chain, the molecule is internalized and the A chain removes a single adenine from the 28S rRNA of the ribosome, preventing further binding of elongation factors. This leads to an arrest of protein synthesis and finally results in cell death. Different *in vitro* assays have been developed to detect the functional activity of ricin, among them assays that detect the glycan-binding capacity of the B chain [88] or assays that detect the isolated enzymatic activity such as adenine release assays or cell-free translation assays based on rabbit reticulocyte lysate [43,89,90]. Since the detection of the activity of the isolated subchains provides only limited information on the activity of the intact ricin molecule, assays for both subchains are required for the detection of active ricin. In this context, cytotoxicity assays have found broad application, ranging from end-point assays to real-time cytotoxicity formats [45].

**Figure 11:**
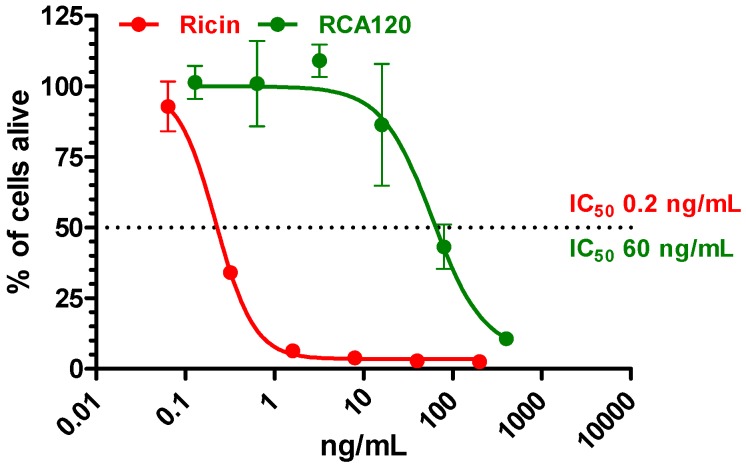
Real-time cytotoxicity assay to display the cytotoxic activity of the purified ricin and RCA120 preparations. Vero cells were seeded in a 96-well E-plate at 12,500 cells/well; immediately after seeding, cells were exposed to different concentrations of ricin (red) or RCA120 (green). Cell proliferation was dynamically monitored over 36 h using an xCelligence instrument (OMNI Life Science GmbH & Co, Bremen, Germany [45]). For calculation of the IC_50_ values of ricin and RCA120, the percentage of cells alive was plotted against the concentration of ricin (red) or RCA120 (green) at 36 h. Cell index of cells without treatment at 20 h was set to 100%. Calculated IC_50_ for ricin was 0.2 ng/mL and for RCA120 60 ng/mL. One representative experiment out of two performed in triplicate is shown.

In this work, a real-time cytotoxicity assay based on impedance measurement was used to characterize both the purified ricin and the purified RCA120 preparation [45]. The cell proliferation and toxin-induced cell death of African green monkey (Vero) cells was monitored online using the xCELLigence system (OMNI Life Science GmbH & Co. KG, Bremen, Germany). This system is based on an impedance sensor technology to quantify cell viability and proliferation noninvasively and without labeling. Cell proliferation, morphology, and adhesion are monitored in real time. Cells are seeded into E-plates with integrated microelectrodes. Upon low-voltage application an electric field is generated which is differentially modulated by the cells. The higher the number of cells attached to the plate surface, the higher the impedance monitored by the instrument is. This change in impedance is used as a read-out for cell viability [45].

Using a standardized protocol, Vero cells were incubated over 36 h with increasing concentrations of purified ricin or purified RCA120. *In vitro* cytotoxicity, where 50% of the cells were alive (IC_50_), was calculated after 36 h by displaying the percentage of cells alive against the concentration of the lectin used in the test (Figure 11). For ricin, an IC_50_ of 0.2 ng/mL was calculated, while for RCA120, an IC_50_ of 60 ng/mL was determined. Based on these data, ricin is approximately 300-fold more toxic than RCA120 in the experimental system used. While it is well established that RCA120 is much less toxic than ricin, the difference in activity depends on the type of assay as well as on the purity and integrity of the lectin preparations used; differences in activity from 60-fold to 2000-fold can be found in the literature [8,22,23,24].

## 3. Experimental Section

### 3.1. Purification of Ricin and RCA120 and Determination of Protein Concentration

Ricin and RCA120 were purified from the seeds of *R. communis* variety *carmencita pink* following protocols similar to those described earlier [8,45,46]. Briefly, the purification process consisted of an acid extraction at pH 4 and an ammonium sulfate precipitation of proteins. Affinity separation using an in-house prepared lactosyl-Sepharose-4B column was used to isolate ricin and RCA120 from other constituents in the precipitate. Finally, size exclusion chromatography over HiLoad Superdex 200 prep grade (General Electrics, Health Care Life Sciences, Uppsala, Sweden) was used to separate ricin from RCA120.

For determination of the precise concentration of the purified ricin and purified agglutinin preparations, the absorption at 280 nm, 260 nm, and 320 nm were measured in four independent dilutions using a NanoPhotometer (Implen, Munich, Germany) on two different days. Each dilution was measured five times. PBS was used as diluent and as blank reference. The average of the absorption values of each dilution was used to calculate the concentration of both toxin preparations according to Lambert-Beer’s law:

$$c=\frac{A280}{\varepsilon }\times DF\times d$$

with *c*: concentration; A280: absorption at 280 nm; ε: extinction coefficient; DF: dilution factor; *d*: path length of cuvette and the following extinction coefficients: ε (ricin) = 1.1615 mL × mg^−1^ × cm^−1^ (experimentally determined by *Russmann et al.* [52]), which was highly similar to ε (ricin) = 1.17 mL × mg^−1^ × cm^−1^ determined earlier and ε (agglutinin) = 1.17 mL × mg^−1^ × cm^−1^ [6].

### 3.2. SDS-PAGE

Ten percent SDS gels were run in a Mini-PROTEAN^®^ 3 System (Bio-Rad Laboratories, Munich, Germany). Proteins were separated either under non-reducing conditions or under reducing conditions using β-mercaptoethanol (β-ME, SERVA Electrophoresis GmbH, Heidelberg, Germany) as reducing agent, followed by staining with Coomassie Brilliant Blue G250 (SERVA Electrophoresis GmbH, Heidelberg, Germany).

### 3.3. Capillary Gel Electrophoresis

A Bio-Rad Experion electrophoresis instrument (Bio-Rad Laboratories, Hercules, CA, USA) was used with Pro260 chips and the manufacturer’s reagent kit. Four µL of sample were mixed with the sample buffer and analyzed according to the manufacturer’s protocol.

### 3.4. LC-ESI MS for Determination of Molecular Weight

The LC-ESI MS measurements were carried out using the Thermo Finnigan LXQ linear ion trap mass spectrometer (Thermo Scientific, Waltham, MA, USA). The LC separation was done with the Thermo Finnigan Surveyor liquid chromatograph (Thermo Scientific, Waltham, MA, USA) using Supelco Discovery BIO Wide Pore C5 column (Sigma-Aldrich, Bellefonte, PA, USA) (100 × 2.1 mm, 3 µm particles, 300 Å pore size).

The LC separation was done using a gradient program: 0–7 min 5%–50% B, 7–16 min 50%–95% B, 16–21 min 95% B, 21–22 min 95%–5% B, 22–32 min 5% B, where eluent A is ultra high quality water (reverse osmosis and ion exchange) with 0.1% of formic acid, and B is acetonitrile with 0.1% of formic acid. The injection volume was 10 µL.

The mass range for the measurements was 400–2000 *m*/*z* and the spectra were measured in the profile mode. The voltage of the ESI needle was 4 kV. The ion source capillary was kept at 290 °C and at 42 V. Sheat gas was kept at 20 (arbitrary units) and Aux gas at 4 (arb). Deconvolution was done using ProMass for Xcalibur (version 2.8, rev. 2, Novatia LLC, Newtown, PA, USA).

### 3.5. LC-ESI MS for Tryptic Fingerprint and Sequencing

For LC-MS analysis aliquots of purified ricin and purified RCA120 were digested with trypsin after reduction and alkylation according to published procedures [44]. Sample preparation and digestion was performed directly in a centrifugal cartridge with a 10 kDa molecular weight cut-off membrane (Microcon YM-10, Merck Millipore, Billerica, MA, USA). Briefly, 50 µL of the sample were transferred to the cartridge and centrifuged at 14,000× *g* until approximately 10 µL remained. The reducing solution, 200 µL of 3 mg DTT/mL in guanidine buffer (6 M guanidine HCl in 0.1 M Tris pH 8) was added and the sample was reduced at 60 °C for 1 h. Then, 67 µL of 7.5 mg iodoacetamide/mL in the guanidine buffer was added and the sample was alkylated at 37 °C in the dark for 30 min. The reduced and alkylated sample was centrifuged to approximately 10 µL and washed twice with 200 µL of 0.1 M ammonium bicarbonate. The digestion buffer, 200 µL of 0.1 M ammonium bicarbonate, and 0.5 µg of modified trypsin (Promega, sequencing grade) was added and the sample was digested for 6 h at 37 °C before centrifugation to collect the digest peptides. The digests were stored at −20 °C until analysis.

A Waters nano-Acquity-Q-Tof Ultima LC-MS system equipped with a nanoflow electrospray ionization source was used for sample analysis (Micromass, Altrincham, UK). Digests were diluted 1:50 in 0.4% formic acid containing 5 fmol/µL of leucine-enkephalin internal standard. A 2-µL sample was injected and trapped on a C18 trap column which was subsequently eluted onto a Waters BEH130 C18 75 µm in diameter, 150 mm column with a 3%–60% acetonitrile gradient containing 0.1% formic acid at 400 nL/min. The mass scale was calibrated using sodium formate clusters. Mass spectra were acquired over the mass range 300–1800 at 1 scan/s. Precursor ions were selected with a 3 Da mass window, and product ion spectra were recorded from 100 and up to a mass appropriate for each of the precursor ions.

### 3.6. MALDI TOF-MS

First, 15.1 µg of ricin and 10.3 µg of RCA120 were separated on a non-reducing 10% SDS-PAGE. Coomassie-stained gel bands were cut out with a clean scalpel, divided into equal sections of approximately 1.5 mm size, and transferred into Eppendorf (Hamburg, Germany) LoBind tubes. In order to destain the protein bands, gel pieces were covered twice with 200 µL of 200 mM ammonium bicarbonate/40% acetonitrile for 30 min at 37 °C. Reduction was performed with 15 µL of 400 mM dithiothreitol for 10 min at 95 °C. Alkylation was carried out using 30 µL of 500 mM iodoacetamide at 37 °C for 30 min in the dark. Reduced and alkylated samples were digested with 20 µL of trypsin (0.4 µg) (Sigma-Aldrich, Taufkirchen, Germany, proteomics grade) at 37 °C overnight. Reaction was terminated with 50 µL of 0.1% trifluoroacetic acid/50% acetonitrile, for 30 min at 37 °C. Digested peptides were further desalted and concentrated with ZipTip C18 resin (Merck Millipore, Darmstadt, Germany) carried out according to manufacturer’s instructions.

Sample analysis was done utilizing an autoflex speed MALDI-TOF/TOF mass spectrometer (Bruker Daltonics, Bremen, Germany) with a polished steel MTP 384 target plate (Bruker Daltonics, Bremen, Germany). One µL sample was mixed with 1 µL of α-Cyano-4-hydroxycinnamic acid (12 mg/mL; Bruker Daltonics, Bremen, Germany), and 1 µL was deposited on the target to let it dry. For matrix suppression deflection was set to 600, and mass spectra were acquired over the mass range 600–4000. External calibration was performed with peptide calibration standard II (Bruker Daltonics, Bremen, Germany). Spectra were processed by flexAnalysis 2.4 and BioTools 3.2 software (Bruker Daltonics, Bremen, Germany).

### 3.7. ELISA

The ricin-specific ELISA was performed essentially as described before [70], using mAb antibody R109 as capture antibody and biotinylated R18 as detection tool, followed by a streptavidin-poly-horseradish peroxidase conjugate (PolyHRP40). Briefly, MaxiSorp microtiter plates were coated with primary mAb (10 µg/mL) in 50 µL of PBS overnight at 4 °C and blocked with casein buffer (Senova, Jena, Germany) for 1 h at room temperature. Following washing, 50 µL of toxin was added in serial dilutions from 100 ng/mL to 0.05 pg/mL in assay buffer (PBS, 0.1% BSA (Sigma-Aldrich, Munich, Germany)) and incubated for 2 h at room temperature. The sandwich ELISA was developed by incubation with biotin-labeled secondary antibody diluted in casein buffer (1 h, room temperature), followed by washing and detection with Streptavidin-PolyHRP40 (0.5 ng/mL, Senova, Jena, Germany) and substrate 3,3′,5,5′-tetramethylbenzidine (TMB, Seramun Diagnostica GmbH, Heidesee, Germany).

The RCA120-specific ELISA was performed similarly, using mAb ARK4 (kindly provided by Marc-André Avondet, Spiez Laboratory, Switzerland; [71]) as capture antibody and biotinylated polyclonal chicken IgY RC22 [72] as detection antibody.

### 3.8. Cytotoxicity Assay

Measurement of the cytotoxicity of ricin and RCA120 was performed by a real-time cytotoxicity assay based on impedance measurement (xCELLigence system, OMNI Life Science GmbH & Co, Bremen, Germany) as described before [45]. Briefly, after baseline measurement of the E-plate, a Vero cell suspension containing 12,500 cells/well in a volume of 75 µL was seeded into the E-plates. Immediately after the seeding of cells into the wells (*i.e.*, without prior attachment of the cells onto the plate), purified ricin or purified RCA120 at concentrations of 0.064 ng/mL to 200 ng/mL for ricin or 0.128 ng/mL to 400 ng/mL for RCA120 were added onto the cells in a volume of 30 µL. Each sample was measured at least in duplicate. The cell index was automatically determined every 15 min by the xCELLigence system over a period of up to 36 h. During the incubation, only live cells attached onto the plate and showed vigorous proliferation (equivalent to an increase in impedance and cell index). Depending on the toxin concentration present on the cells, the proliferation was terminated after different time points, followed by detachment and cell death (equivalent to a drop of impedance and cell index). Cell viability was converted into percent (%) of the control cells alive over toxin concentration. To this end, the cell index of nontreated cells measured at several time-points was set to 100%, and for a given time-point the ratio of cell index values of toxin-treated cells to untreated cells was calculated. *In vitro* cytotoxicity at 50% (IC_50_) was defined as the toxin concentrations required to reduce cell viability by 50% compared to untreated control cells [45].

## 4. Conclusions

Although different technologies for the detection and identification of the plant toxin ricin as a potential biological weapon under the BWC and CWC have been established worldwide, hardly any universally agreed-upon “gold standards” are available, *i.e.*, common internationally recognized reference materials. The current situation is that expert laboratories use differently purified in-house materials of variable quality for their own validation purposes, making any comparison of accuracy and sensitivity of different methods nearly impossible. The current work has been undertaken in an effort to generate highly pure and well-characterized materials that can be used as a starting point to develop certified reference materials.

In this work, the plant toxin ricin and the highly homologous *R. communis* agglutinin RCA120 were purified from the seeds of *R. communis* variety *carmencita pink* to a purity of >97% and >99%, respectively. Both protein preparations were extensively characterized using biochemical, spectrometric, immunological, and functional approaches combining technical expertise available in different European laboratories in the framework of the EU-funded project EQuATox. Based on the in-depth characterization performed in different European expert laboratories, the protein preparations represent reference materials of high quality which were later used in an international proficiency test [42]. Indeed, in this exercise a consensus concentration was determined for the ricin reference material based on different technological approaches available in independent expert laboratories, indicating that the comparability of results was achieved [42].

The employed mass spectrometric analyses on tryptically digested materials revealed peptides suitable for the identification and quantification of ricin isoforms and RCA120, *i.e.*, peptides that are proteotypic and detected at high intensity, and our results are in good agreement with previously published data obtained with independent or commercial ricin preparations of different quality. Recommendations are given as to which and how many peptides should be used to identify ricin D, ricin E, and RCA120 unambiguously, and this information is potentially important in the course of a forensic investigation.

As screening methods, two different ELISA methods were presented that preferentially detect either ricin or RCA120 with only very little cross-reactivity between the two homologous analytes down to the low pg/mL range, a concentration range that is relevant in the context of human ricin intoxications (own unpublished data and [91]). While the ELISA-based methods are not suitable for the unambiguous identification of ricin, they are most sensitive to detect ricin-containing samples in the low pg/mL range which is usually not covered by spectrometric approaches. Also, the applied mAb can effectively be used for immunoaffinity enrichment of ricin from complex matrices followed by MS-based identification, combining and improving both technical approaches [62,92,93]. Generally, it has to be considered that antibody binding might be affected by the glycosylation of ricin/RCA120, and interference could occur if the epitope recognized by an antibody contains or is adjacent to a specific glycosylation site. However, when the ricin-ELISA described in this work was used to detect ricin prepared from seven different cultivars of *R. communis*, no differences were observed when equivalent amounts of toxin were tested (not shown).

In terms of function, a real-time cytotoxicity assay showed that the ricin reference material prepared was approximately 300-fold more toxic than the corresponding RCA120 preparation, with an IC_50_ value of 0.2 ng/mL for ricin and 60 ng/mL for RCA120. Exemplarily, previously published IC_50_ values for ricin were determined at 8–60 ng/mL [29] and 0.14–0.4 ng/mL [45,94] and showed that the precise IC_50_ value depends on different analytical parameters such as the cell line used for the cytotoxicity assay, kinetics, purity, integrity, and post-translational modification of the toxin used. Evaluation of toxicity in mice provided evidence for ricin being about 40–80-fold more toxic than RCA120 [24], but comparative data on non-human primates are not available [95]. Overall, comparison of the toxicity data is difficult as often no detailed information on the purity, lectin composition, or the cultivar used is available. On the other hand, rodent and non-human primate models have been valuable to delineate the pathophysiology of ricin toxicosis after systemic, oral, or aerosol exposure or to analyze the efficacy of potential therapeutic agents [96].

In the context of protein function it would be interesting to extend the characterization further to i) the identification of glycosylation patterns of the ricin isoforms isolated; ii) the precise quantification of constituent ricin isoforms (D and E) in the mixture; and iii) the elucidation of secondary structures to highlight and follow the integrity of the molecules over time (e.g., by using circular dichroism spectroscopy). Finally, it might be interesting to compare results obtained with ricin reference material isolated from different cultivars of *R. communis* for additional validation of identification and quantification methods. Especially glycosylation has been shown to be important for protein function, and different glycosylation correlates with different toxicities [29]: of three differently glycosylated ricin isoforms tested, the highest glycosylated form, containing more hybrid/complex-type glycans with mannose as hexose units, has been shown to be most toxic in different assay systems. Along the same line, the chemically deglycosylated ricin A chain is about 1000-fold less toxic than the glycosylated ricin A chain in mice [97]. Furthermore, *N*-glycosylation has been shown to promote the toxicity of the ricin A chain by promoting its transport out of the endoplasmic reticulum [98]. Overall, there is convincing evidence showing that glycosylation and especially the type of glycosylation of ricin does affect the function of the molecule, a fact that is well known for a range of different proteins [99]. This is currently seen as a counter-argument against the production of ricin reference material as a recombinant protein in prokaryotic or eukaryotic expression systems (e.g., *E. coli*, *P. pastoris* or tobacco plants), which was a successful approach for vaccine development [100]. To have the authentic glycosylation pattern or post-translational modification, in general, on a ricin reference material purified from natural sources seems to outweigh advantages of recombinant technologies such as the higher uniformity of proteins produced. However, recombinant technologies or, in light of the cellular toxicity of ricin, cell-free expression systems could be the way forward to produce stable isotope-labeled ricin that could be used in the analytical workflow to determine sample preparation recovery rates or to document the reproducibility of analytical procedures (e.g., enzymatic digestion efficiency for spectrometric approaches). A prerequisite for using this approach, however, is that the pure (nonglycosylated) isotopically labeled protein behaves the same way as the authentic protein.

Taken together, the experience acquired in this study can be exploited further in a next step to develop certified ricin and RCA120 reference materials that would be available to international expert laboratories. To place the activities in a larger context, decision-makers rely on correct and reliable laboratory data in order to make appropriate decisions. Reliable detection, identification, and quantification of ricin and RCA120 as well as their discrimination requires highly pure and well-characterized reference materials. Due to the fact that ricin is classified as a C-weapon, this issue could well be driven forward by internationally funded projects with technological expert support from different nations and standardization bodies. Based on this, the cornerstones of the next developments in the process of harmonization of analytical approaches would be highly specific tools, thoroughly validated analytical procedures, recommended standard operating procedures, well-trained personnel, and regular training in proficiency tests or ring trials.

## Supplementary Materials

Supplementary materials can be accessed at: http://www.mdpi.com/2072-6651/7/12/4856/s1.
