# Supplementary material for: Characterization of Ricin and R. communis Agglutinin Reference Materials
Source: Toxins (Basel). 2015 Nov 26;7(12):4906–34. doi: 10.3390/toxins7124856 (PMC4690106; doi:10.3390/toxins7124856)
Supplement: Supplementary file 1 [file toxins-07-04856-s001.pdf]

Supplementary Information

(a)

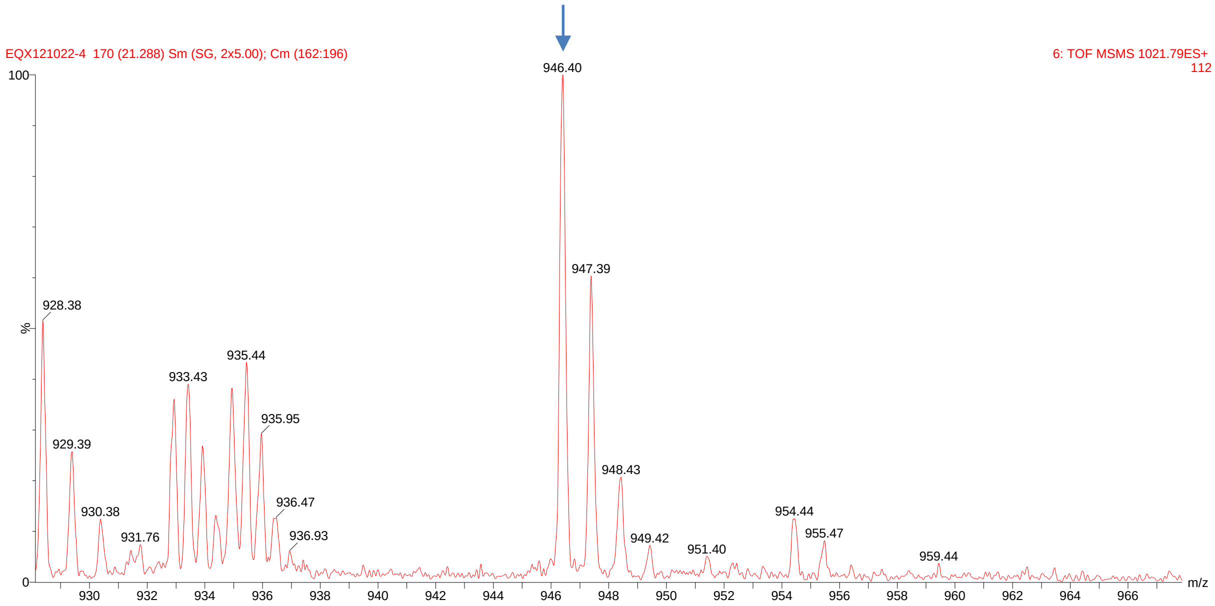

T10b: CLTTYGY**SP**GVYVMIYDCNTAATDATR: b8 ion at m/z 946.40^{+}

(b)

a	668.31	831.37	918.40	1015.46	1072.48	1171.55
b	696.30	859.37	946.40	1043.45	1100.47	1199.54
	6	7	8	9	10	11
	Gly	Tyr	Ser	Pro	Gly	Val
	22	21	20	19	18	17
y"	2425.08	2368.06	2205.00	2117.96	2020.91	1963.89

a	668.31	831.37	928.42	1015.46	1072.48	1171.55
b	696.30	859.37	956.42	1043.45	1100.47	1199.54
	6	7	8	9	10	11
	Gly	Tyr	Pro	Ser	Gly	Val
	22	21	20	19	18	17
y"	2425.08	2368.06	2205.00	2107.94	2020.91	1963.89

Figure S1. (**a**) Expanded product ion spectrum of T10b (*m/z* 1021.79^{3+}) from the trypsin digest of the purified ricin sample. The Ser-Pro sequence as in ricin D is consistent with the ion at *m/z* 946.40 (arrow) and the doubly charged ion at *m/z* 1059.50^{2+} (2117.99^{+}) resulting from cleavage of the Ser-Pro bond (the b8 and y19 sequence ions, respectively). A Pro-Ser sequence would give a (weak) b8/y19 pair at 956.52/1054.47^{2+}. The Pro-Ser as in the database sequence GI:225419 of ricin E could not be confirmed in the purified ricin sample; (**b**) The theoretical masses of the sequence ions around the Ser70-Pro71 of GI:225896, GI:2169612 (ricin E) and GI:132567 (ricin D) (upper part) and Pro70-Ser71 of GI:225419 (lower part).

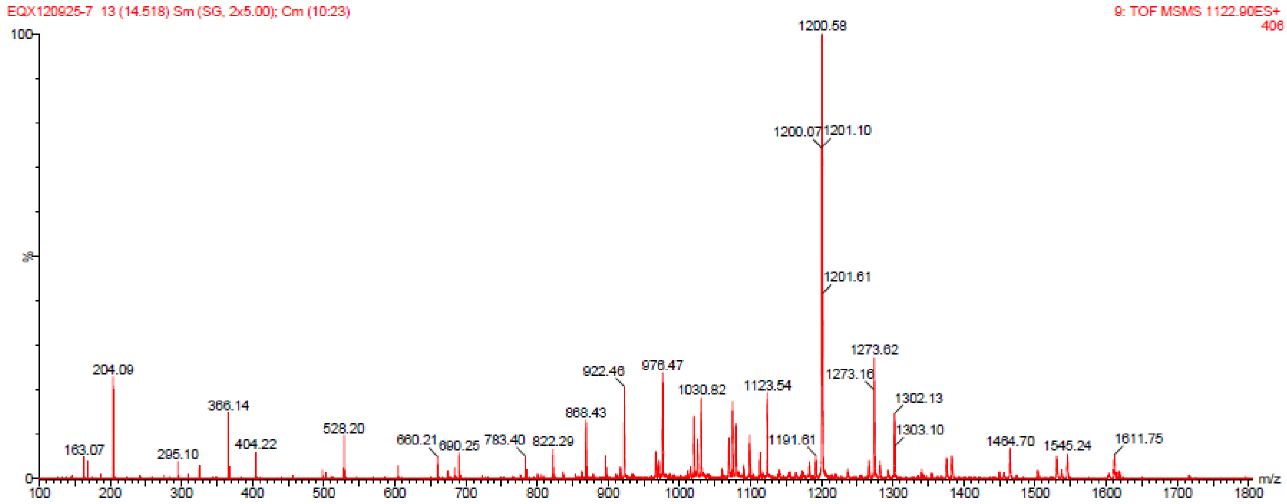

T9b: SSPGQQVVIYNCSTATVGATR m/z 1122.84^{3+}

a	60.04	147.08	244.13	301.15	429.21	557.27	656.34	755.41	868.49	1031.55	1145.60
b	88.04	175.07	272.12	329.15	457.20	585.26	684.33	783.40	896.48	1059.55	1173.59
	1	2	3	4	5	6	7	8	9	10	11
	**Ser**	**Ser**	**Pro**	**Gly**	**Gln**	**Gln**	**Val**	**Val**	**Ile**	**Tyr**	**Asn**
	21	20	19	18	17	16	15	14	13	12	11
y"	-	2109.04	2022.01	1924.95	1867.93	1739.88	1611.82	1512.75	1413.68	1300.60	1137.53

a	1305.63	1392.66	1493.71	1564.74	1665.79	1764.86	1821.88	1892.92	1993.97	-
b	1333.62	1420.65	1521.70	1592.74	1693.79	1792.85	1849.88	1920.91	2021.96	-
	12	13	14	15	16	17	18	19	20	21
	**CAM**	**Ser**	**Thr**	**Ala**	**Thr**	**Val**	**Gly**	**Ala**	**Thr**	**Arg**
	10	9	8	7	6	5	4	3	2	1
y"	1023.49	863.46	776.43	675.38	604.34	503.29	404.23	347.20	276.17	175.12

Figure S2. LC-MS product ion spectrum of the RCA120 T9b glycopeptide diagnostics for the RCA120 isoform corresponding to database sequence GI:225114. For sequencing the RCA120 T9b ion (*m/z* 1122.84^{3+}) was extracted at a retention time of 14.50 min.

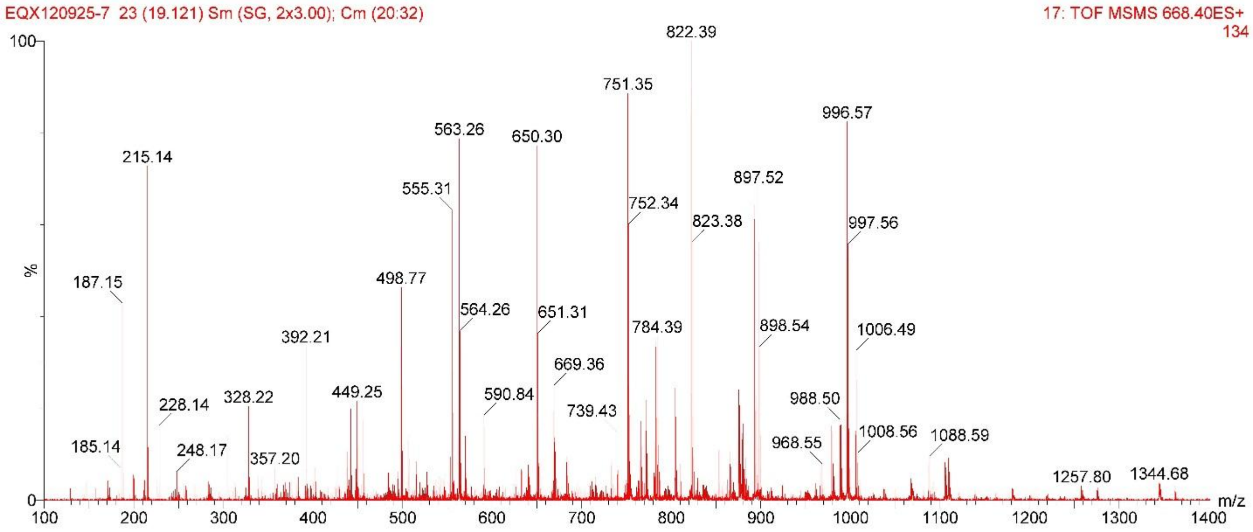

T11b: TIINPTSGLVLAATSGNSGTK m/z 668.0320^{3+}

a	74.06	187.14	300.23	414.27	511.32	612.37	699.40	756.43	869.51	968.58	1081.66
b	102.06	215.14	328.22	442.27	539.32	640.37	727.40	784.42	897.50	996.57	1109.66
	1	2	3	4	5	6	7	8	9	10	11
	Thr	Ile	Ile	Asn	Pro	Thr	Ser	Gly	Leu	Val	Leu
	21	20	19	18	17	16	15	14	13	12	11
y"	-	1901.03	1787.95	1674.87	1560.82	1463.77	1362.72	1275.69	1218.67	1105.59	1006.52

a	1152.70	1223.74	1324.78	1411.82	1468.84	1582.88	1669.91	1726.93	1827.98	-
b	1180.69	1251.73	1352.78	1439.81	1496.83	1610.88	1697.91	1754.93	1855.98	-
	12	13	14	15	16	17	18	19	20	21
	Ala	Ala	Thr	Ser	Gly	Asn	Ser	Gly	Thr	Lys
	10	9	8	7	6	5	4	3	2	1
y"	893.43	822.40	751.36	650.31	563.28	506.26	392.21	305.18	248.16	147.11

Figure S3. LC-MS product ion spectrum of RCA120 T11b peptide diagnostics for the RCA120 isoform corresponding to database sequence GI:225114. For sequencing the RCA120 T11b ion (*m/z* 668.032^{3+}) was extracted at a retention time of 19.10 min.

(a)

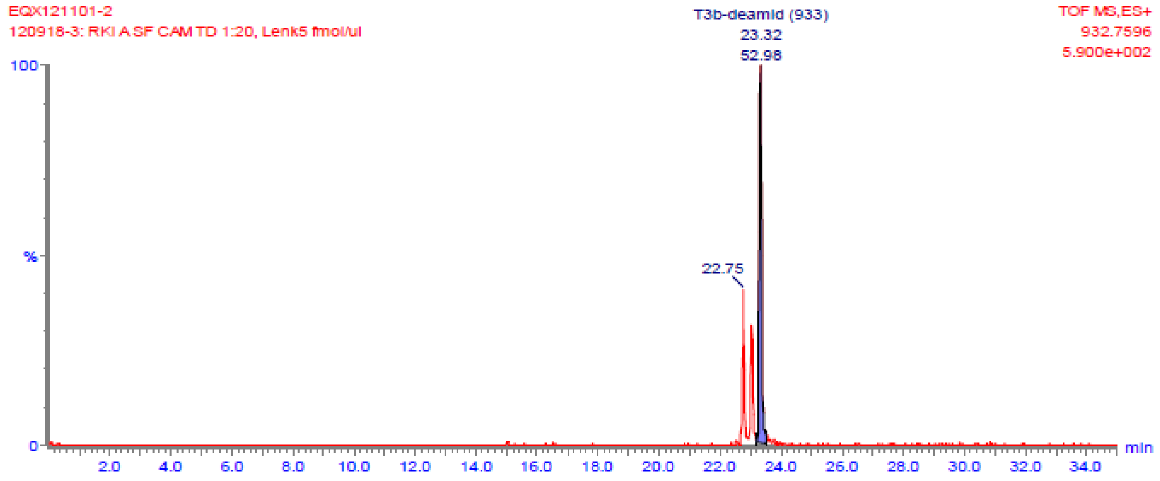

(b)

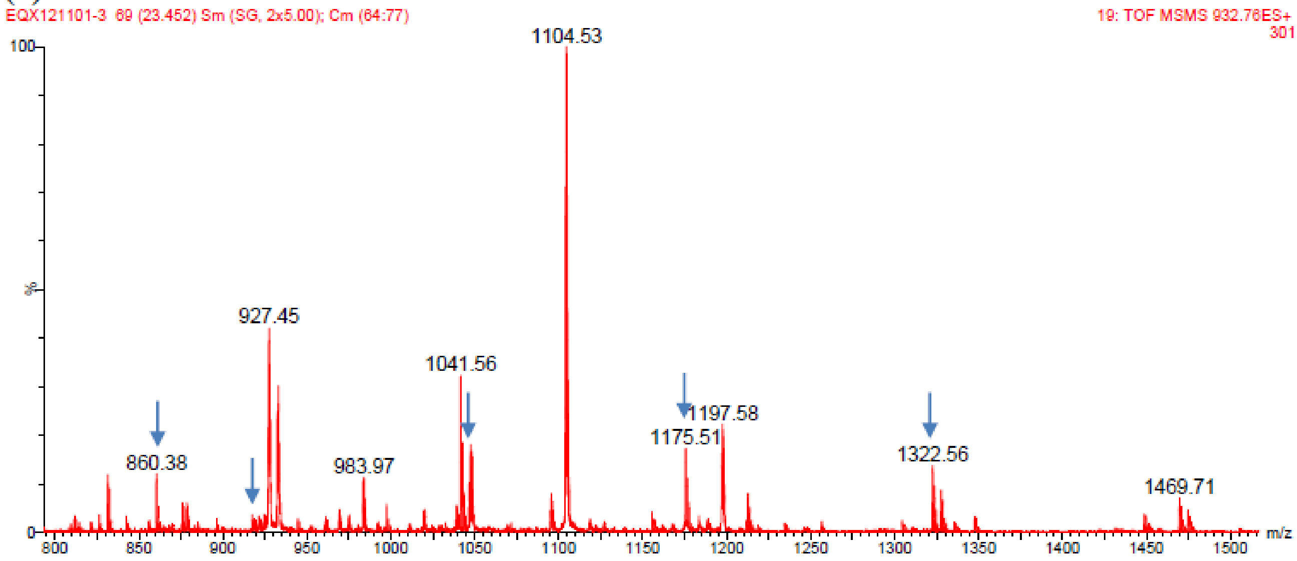

a	731.34	832.39	889.41	1018.45	1147.49	1294.56	1441.63	1556.66
b	759.33	860.38	917.40	1046.45	1175.49	1322.56	1469.63	1584.65
	7	8	9	10	11	12	13	14
	Val	Thr	Gly	Glu	Glu	Phe	Phe	Asp
	18	17	16	15	14	13	12	11
y"	2137.01	2037.94	1936.89	1879.87	1750.83	1621.78	1474.72	1327.65

Figure S4. LC-MS extracted ion chromatogram and product ion spectrum of RCA120 T3b (<u>D</u>GLCVDV**T**GEEF**F**DGNPIQLWPCK *m/z* 932.7596^{3+}). (**a**) The peak at 22.75 min in the extracted ion chromatogram corresponds to the native peptide and the peaks at 23.03 and 23.32 min to the deamidated peptide containing an aspartic acid and iso-aspartic acid, respectively. The Asn at the N-terminal is prone to deamidation which in this case has occurred mainly during sample preparation [30]; (**b**) Product ions b8–b12 in the product ion spectrum (blue arrows) support the –**T**GEEF**F**– sequence in RCA120 GI:113504 instead of –**F**GEEF**T**– in the RCA120 sequence GI:225114.

Results and Discussion

For the chain B of RCA120 two different protein sequences are available in the databases, GI:225114 and GI:113504 (the latter corresponding to Uniprot P06750, X-ray structure: 1RZO) which differ in six amino acid positions (aa 24, 29, 60, 72, 102 and 250).

With respect to our purified RCA120 preparation, the experimentally found protein sequence of the T3 peptide of chain B matches with the sequence of the X-ray structure (Uniprot P06750 and structure 1RZO; aa in positions 24 and 29 were identified as T^{24} and F^{29}).

On the other hand, the experimentally determined T9 glycopeptide, T11 and T22 peptides of the RCA120 chain B sequence differ from the sequence of the X-ray structure in three amino acid positions (aa 72, 102 and 250, experimentally identified were G^{72}, T^{102}, V^{250}; please note, that position 60 was not covered by protein sequencing), and these results are in agreement with GI:225114.

It is unclear if the difference in aa positions between GI:225114 and GI:113504 have a critical influence on the overall three dimensional structure depicted in 1RZO.
